# Short cell-penetration peptide conjugated bioreducible polymer enhances gene editing of CRISPR system

**DOI:** 10.1186/s12951-024-02554-w

**Published:** 2024-05-24

**Authors:** Xiaobo Wang, Chengyuan Cai, Weiqi Lv, Kechen Chen, Jiaxin Li, Kaitong Liao, Yanqun Zhang, Hongxin Huang, Ying Lin, Zhili Rong, Xiaopin Duan

**Affiliations:** 1 Dermatology Hospital, Southern Medical University, Guangzhou, 510091 China; 2 Cancer Research Institute, School of Basic Medical Sciences, State Key Laboratory of Organ Failure Research, National Clinical Research Center of Kidney Disease, Key Laboratory of Organ Failure Research (Ministry of Education), Southern Medical University, Guangzhou, 510515 China; 3 Guangzhou Key Laboratory for Research and Development of Nano-Biomedical Technology for Diagnosis and Therapy and Guangdong Provincial Education Department Key Laboratory of Nano-Immunoregulation Tumor Microenvironment, Department of Oncology and Translational Medicine Center, The Second Affiliated Hospital, Guangzhou Medical University, Guangzhou, 510260 China; 4 Experimental Education/Administration Center, School of Basic Medical Science, Southern Medical University, Guangzhou, 510515 China

**Keywords:** CRISPR system, Bioreducible polymer, Short cell-penetration peptide, Transcriptional activation, Genome editing

## Abstract

CRISPR-based gene therapy offers precise targeting and specific editing of disease-related gene sequences, potentially yielding long-lasting treatment effects. However, efficient delivery remains a significant challenge for its widespread application. In this study, we design a novel short peptide-conjugated bioreducible polymer named TSP^scp^ as a safe and effective delivery vector for the CRISPR system. Our results show that TSP^scp^ markedly boosts transcriptional activation and genome editing activities of multiple CRISPR systems as confirmed by decomposition-seq and Deep-seq, which is resulted from its capability in facilitating delivery of plasmid DNA by promoting cellular uptake and lysosomal escape. Additionally, TSP^scp^ further enhances genome editing of CRISPR by delivery of minicircle DNA, a condensed form of regular plasmid DNA. More importantly, TSP^scp^ significantly improves delivery and genome editing of CRISPR system in vivo. In summary, our study highlights TSP^scp^ as a promising delivery tool for CRISPR applications in vivo.

**Graphical Abstract:**

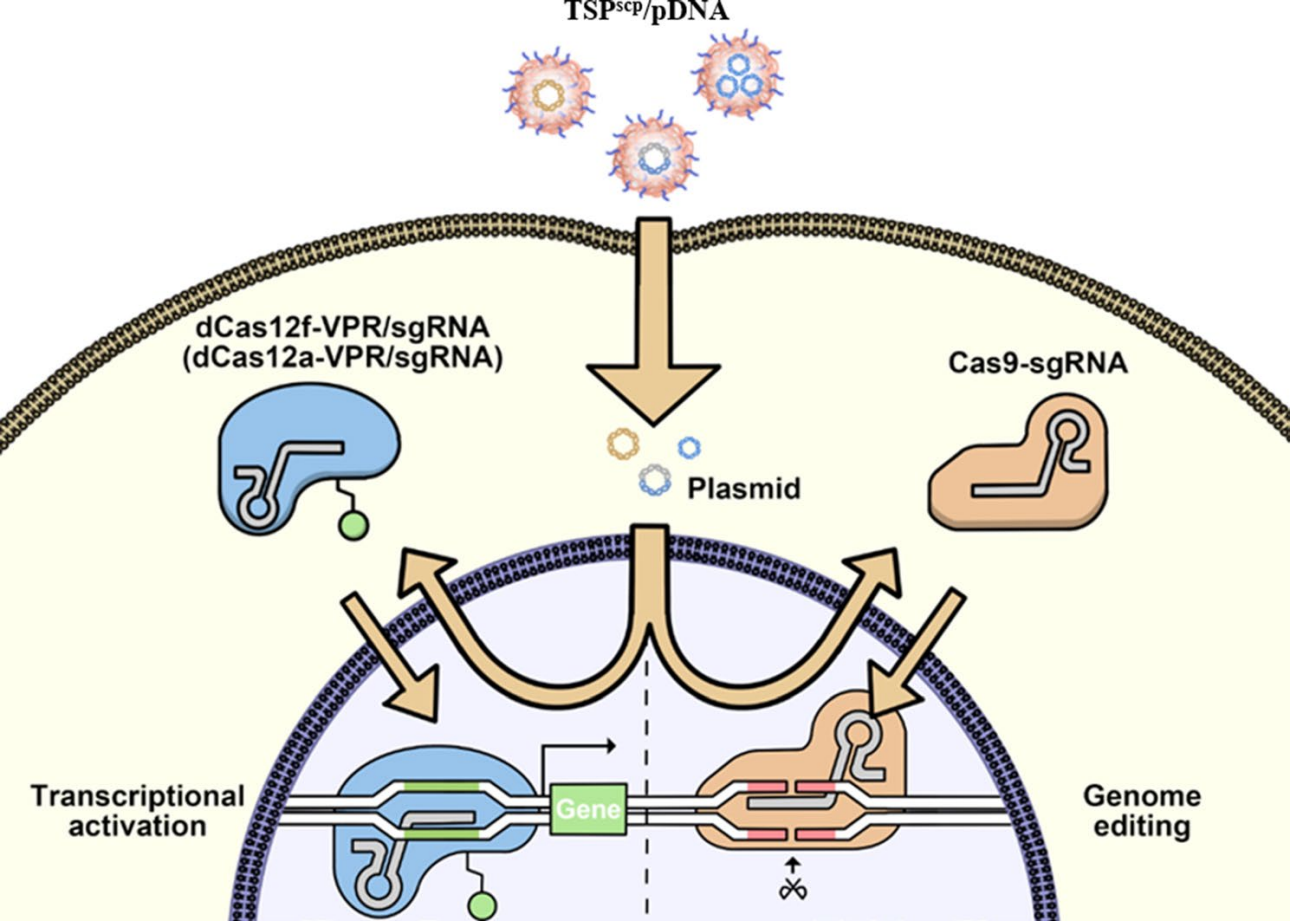

**Supplementary Information:**

The online version contains supplementary material available at 10.1186/s12951-024-02554-w.

## Introduction

CRISPR-Cas systems have revolutionized targeted editing in human cells [1–4], opening up new possibilities in treatment of human diseases [5, 6]. CRISPR-based gene therapy can offer precise targeting and specific editing of disease-related gene sequences, potentially providing long-lasting treatment effects [7–9]. Notably, the U.S. Food and Drug Administration (FDA) recently approved the first CRISPR-based treatment for sickle cell disease, marking a significant milestone in the field [10]. However, challenges persist in CRISPR-based gene therapy, with one key hurdle being the achievement of efficient delivery to targeted cells [11, 12]. While both viral vectors, like adeno-associated viral vectors [13, 14], and non-viral vectors, such as lipid nanoparticle [15–17] and cationic polymers [18, 19], have been utilized for CRISPR delivery, the effectiveness of these delivery systems continues to face challenges due to the pre-existing immunity risks, cargo size limitations, and safety concerns [20].

Plasmid DNA (pDNA) is an exceptional vector for CRISPR-based gene therapy due to its inherent stability, ease of large-scale preparation, and cost-effectiveness [21]. However, CRISPR pDNA must overcome various physiologic barriers to exert its functions, including cell entry, endosome escape, nuclear translocation, transcription, and post-transcriptional activation, all of which necessitate efficient delivery vectors. Cationic polymers, such as polyethyleneimine (PEI), are commonly employed in gene delivery because of their capacity to shield nucleic acid cargos, boost cellular uptake, and aid in lysosome escape [22]. Nevertheless, high molecular weight PEI (e.g., 25 kDa) can enhance transfection efficiency at the expense of increased cytotoxicity. Consequently, derivatives with low molecular weight PEI (e.g., 2 kDa or 800 Da) have been engineered to address the balance between transfection efficiency and cellular toxicity [23]. Furthermore, the incorporation of cell-penetration peptides offers a strategy to enhance transfection by facilitating the cellular entry of macromolecules [24, 25].

**Fig. 1:**
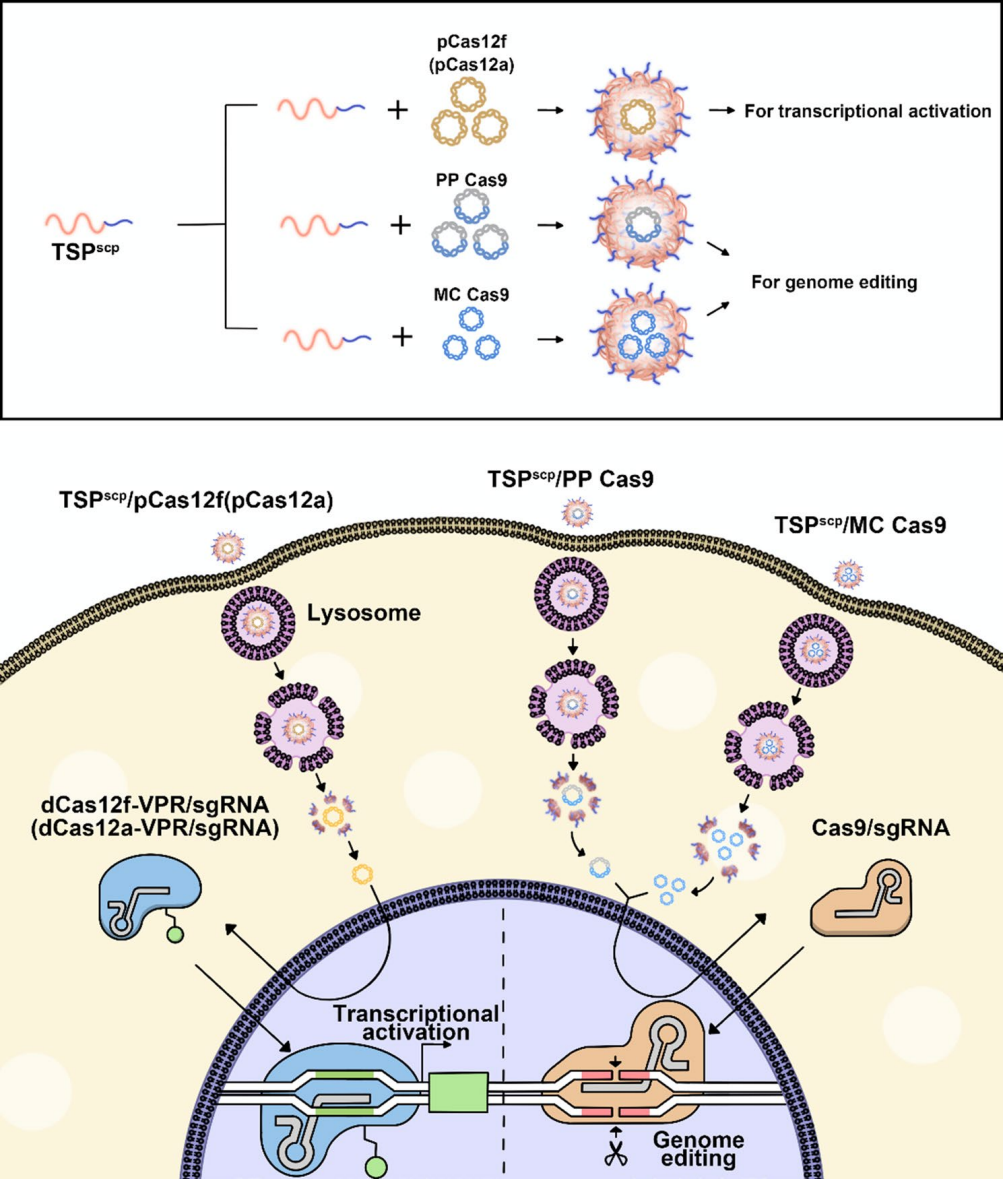
Schematic illustration of TSP^scp^ facilitating efficient cellular uptake of CRISPR pDNA. pCas12f, plasmid DNA expressing mcherry-dCas12f-VPR/sgRNA (VPR, VP64-p65-Rta, transcriptional activator). pCas12a, plasmid DNA expressing mcherry-dCas12a-VPR/sgRNA. PP Cas9, parental plasmid DNA expressing Cas9. MC Cas9, minicircle DNA expressing Cas9

In this study, we developed a PEI derivative called TSP^scp^ to efficiently transfect the CRISPR pDNA (Fig. 1). TSP^scp^ was created by conjugating Tween 85 to low molecular weight PEI 2 K via a bioreducible disulfide linkage and integrating a short cell-penetrating peptide (SCP) with the sequence AC-TGSTQHQ-CG. SCP peptide has been demonstrated to enhance uptake of siRNA in skin cells [26]. Furthermore, our previous research (unpublished data) revealed that SCP also improved the cellular uptake of plasmid DNA in HEK293T cell line. PEI 2 K was selected for its low cytotoxicity, while Tween 85 was utilized to enhance cellular uptake and improve transfection efficacy by interacting with the low-density lipoprotein receptor [23]. TSP^scp^ formed spherical nanocomplexes with CRISPR pDNA through electronic interactions, safeguarding the DNA from degradation, enhancing internalization, enabling lysosomal escape, and promoting nuclear translocation in a bioreducible-responsive manner. This delivery system significantly improved gene activation and genomic editing in vitro compared to commercial gene transfection reagents. Furthermore, condensing CRISPR pDNA into smaller minicircle DNA (MC DNA) by removing the bacterial backbone enhanced genome editing efficiency. More importantly, when TSP^scp^ was combined with MC DNA, it also facilitated genome editing in vivo following local injection into mouse skin. Overall, our findings underscore the potential of TSP^scp^ as a promising tool for delivering the CRISPR system in the treatment of skin diseases.

## Materials and methods

### Materials

All plasmids used in this study were listed in Supplementary Table S1. Plasmid pmNG was prepared for TSP^scp^/pDNA characterization. Plasmids of pCas12f containing dUn1Cas12f-VPR fusion and pCas12a containing dLbCas12a-VPR fusion were used for gene activation. Plasmid pCas9 (Addgene #80975), parental plasmids of PP Cas9 or PP Cas9-Cre, and minicircle DNAs of MC Cas9 or MC Cas9-Cre were used for genome editing. The PP Cas9 expressing 3xHA-Cas9 and PP Cas9-Cre expressing Cas9-Cre were constructed by standard PCR and Gibson Assembly. Plasmids expressing single guide RNA (sgRNA) were constructed by ligating the corresponding annealed oligos to the basic plasmid under the human U6 promoter (GENEWIZ, China). SgRNA sequences listed in Supplementary Table S2 were designed through https://www.benchling.com/.

Both the PP vector and the E. coli strain ZYCY10P3S2T were kindly provided by Penghui Zhou Lab.SCP (AC-TGSTQHQ-CG, disulfide bridge 2–10) was purchased from Sangon Biotech (Shanghai). Tween 85, PEI 2K, and PEI 25K were purchased from Sigma Aldrich (Shanghai). N-(3-dimethylaminopropyl)-N’-ethylcarbodiimide hydrochloride (EDCI), 4-dimethylamiopryidine (DMAP) and trifluoroacetic acid (TFA) were obtained from Macklin (Shanghai). Ditertbutyl decarbonate, 3,3’-dithiodipropionic acid (DTDP) and DTT were obtained from Aladdin (Shanghai).

### Synthesis of TSP^scp^

TSP was first synthesized as shown in Supplementary Fig. S1. 4.9 g DTDP dissolved in anhydrous acetonitrile was activated by 3.0 g EDCI and 1.9 g DMAP for 2 h at 37 ^o^C. Subsequently, 10.0 g Tween 85 was then added dropwise to avoid the formation of Tween 85-DTDP-Tween 85 and allowed reaction for 48 h, followed by 46.6 g PEI 2 K addition and further reaction for 12 h. The resulting solution was condensed by rotary evaporation and dialyzed against 95% ethanol (MWCO: 2 kDa) for 48 h to remove unreacted raw materials and produced byproducts, such as Tween 85-DTDP- Tween 85. Ethanol was then removed and the product TSP was dried under vacuum. ^1^H NMR (CDCl_3_): 5.34 (m, 6 H), 3.65 (t, 80 H), 1.27 (m, 60 H), 0.88 (t, 9 H).

To obtain TSP^scp^, 2.0 mg SCP and 6.0 mg ditertbutyl decarbonate were dissolved in Mili-Q water and stirred at 0 °C for 12 h, followed by the addition of 6.0 mg EDCI and 9.0 mg DMAP for another 2 h. 6.0 mg TSP was then added and reacted for a further 24 h. After that, 2 mL TFA and 1 drop of Mili-Q water were added and stirred at room temperature for 4 h. The reaction mixture was condensed by rotary evaporation and dialyzed in 95% ethanol for 48 h to remove unreacted regents. The purified TSP^scp^ was then dried under vacuum and kept in − 80 ℃ for further use. ^1^H NMR (CDCl_3_): 7.37 (d, 1H), 6.79 (d, 1H), 5.34 (m, 6 H), 4.26 (m, 3 H), 4.22 (m, 8 H), 3.69 (m, 9 H), 3.65 (t, 80 H), 2.70–2.95 (m, 4 H), 1.30 (d, 3 H), 1.25 (m, 60 H), 0.88 (t, 9 H), 0.82 (t, 6 H).

### Preparation and characterization of TSP^scp^/pDNA complex

1 µL pmNG (200 ng/µL) in pure water was dropwise added to different amounts of TSP^scp^ (10 mg/µL) under vortexing (weight ratios ranging from 0.3:1 to 10:1), followed by incubating at room temperature for 30 min. The complexes were mixed with loading buffer, run in an agarose gel supplemented with Gel Red dye, and subsequently analyzed using a Bio-Rad gel imaging system. The particle size and zeta potential of TSP^scp^/pmNG complex at the weight ratio of 10:1 were determined by DLS using a Zetasizer (Nano ZS90, Malvern, UK). The morphology was observed under transmission electron microscopy (TEM, H-7650, Hitachi, Japan).

For the stability evaluation, TSP^scp^/pmNG complex at a weight ratio of 10:1 was treated with DTT (20 mM) and/or heparin (50 U/mg DNA) at 37 °C for 2 h and then subjected to agarose gel electrophoresis or DLS determination to evaluate the size changes.

To assess DNA protection, 1 µL TSP^scp^/pmNG complex at a weight ratio of 10:1 was incubated with 10 µL DNase digest buffer containing 1 U restriction endonuclease SalI (NEB) at 37 °C in a water bath for 2 h. Subsequently, SalI in the digest buffer was inactivated by incubating at 98 °C for 1 min. The reaction buffer was then treated with DTT (20 mM) and 1 U Heparin at 37 °C for 2 h, followed by mixing with 6x loading buffer and being subjected to DNA gel electrophoresis.

### Cell culture

HEK293T (ATCC, CRL-3216), MCF7 (ATCC, HTB-22), and mNG KI HEK293T cells were cultured in Dulbecco’s Modified Eagle’s Medium (DMEM, Life Technologies) supplemented with 10% FBS (Life Technologies) and 1% penicillin-streptomycin (Life Technologies). All cell lines were maintained at 37 °C in a humidified incubator with 5% CO_2_ to provide optimal growth conditions.

### Animals

The animal experiments were approved by the Southern Medical University Animal Care and Use Committee. Ai14D mice (B6.Cg-Gt(ROSA)-26Sortm14(CAG-tdTomato)Hze/J, #007914) obtained from The Jackson Laboratory, aged 6–8 weeks, were utilized to assess the genome editing efficiency after intracutaneous injection of TSP^scp^/MC vs. TSP/MC complexes.

### Cell transfection

MCF7 and HEK293T cells cultured in 24-well plates (1 × 10^4^ cells/well) were incubated with complexes of TSP^scp^/pDNA (weight ratio of 40:1), TSP/pDNA (weight ratio of 20:1), PEI 25 K/pDNA (PEI/pDNA, weight ratio of 4:1), or Lipo2000/pDNA (Lipo/pDNA, 1.5 µL Lipo per 500 ng DNA) for 8 h, followed by replacing the medium containing the transfection reagents with fresh complete medium. pDNA expressing CRISPR-Cas nuclease and pDNA expressing sgRNA were mixed by weight ratio of 1:1 for experiments to evaluate gene activation or genome editing. Transfection or cellular uptake efficiencies were quantified by flow cytometric analysis (LSR Fortessa, BD Biosciences, USA) 48 h post-transfection.

### Cytotoxicity

MCF7 and HEK293T cells were seeded in 96-well plates at a density of 5 × 10^3^ cells/well and allowed to adhere for 24 h. Subsequently, the cells were exposed to a range of concentrations of TSP^scp^, from 2 to 128 µg/ml, or TSP^scp^/pmNG complexes at different weight ratios, varying from 5:1 to 160:1 for 48 h. Cell viability was assessed using the cell counting kit-8 (CCK-8) assay (GlpBio, USA) following the manufacturer’s instructions.

### Cellular uptake

The FITC-labeled pCas12f pDNA was complexed with Lipo, PEI 25 K, TSP, and TSP^scp^, respectively, at their optimal weight ratios as described in the section of cell transfection. The resulting complexes were then added to 24-well plates containing MCF7 or HEK293T cells at the concentration of 500 ng pDNA/well and incubated for 2 h, 4 h, or 8 h. After the incubation period, the cells were collected, washed twice with PBS, and then assessed using flow cytometry.

### Lysosome escape

MCF-7 and HEK293T cells were seeded on 24 mm^2^ glass coverslips in a 6-well plate and treated with TSP^scp^/pCas12f-FITC, TSP/pCas12f-FITC, PEI/pCas12f-FITC and Lipo/pCas12f-FITC complexes at a concentration of 500 ng pCas9 per well for 8 h. The cells were stained with LysoTracker Red (Solarbio) for 1 h and 4’,6-diamidino-2-phenylindole (DAPI, H3570, Invitrogen) for 3 min, washed with PBS three times, and finally imaged using a confocal laser scanning microscope (CLSM, A1, Nikon) to visualize the cellular uptake and localization of the pCas12f-FITC complexes.

### Quantitative real‑time PCR (qPCR)

The gene activation ability of dCas12a under different transfection conditions in Fig. 5 was determined using qPCR. Total RNA was isolated 48 h after cell transfection and reverse-transcribed into cDNA using the HiScript II Q RT SuperMix Kit (Vazyme). qPCR was conducted on a LightCycler 96 System (Roche) using the Taq Pro Universal SYBR qPCR Master Mix (Vazyme). The 2-step qPCR program consisted of an initial denaturation step at 95 °C for 30 s, followed by 40 cycles of denaturation at 95 °C for 5 s and annealing/extension at 60 °C for 34 s. The relative gene expression of each target gene was quantified using the 2^−ΔΔCt^ method, normalized to the expression of GAPDH. The qPCR primers used for the IL1RN, HBG1 and TTN genes were listed in Supplementary Table S3.

### Tracking of indels by decomposition (TIDE)-seq

Tracking of indels by decomposition (TIDE)-seq was performed to analyze the frequency of insertions and deletions in a pool of cells. The cells were first collected 48 h after transfection by centrifugation at 300 g for 5 min, resuspended in 200 µL sarkosyl lysis buffer per well, and incubated overnight at 50 °C. After incubation, 2 volumes of anhydrous ethanol and 1/5 volume of saturated NaCl were added to the samples, which were then vortexed for 5 min. Finally, the genomic DNA was extracted by centrifugation and resuspended in water for Sanger sequencing of PCR amplicons.

### Deep-seq library construction and result analysis

Genome editing efficacy was also quantified using next-generation sequencing (NGS) of PCR amplicons. Genomic DNA was extracted from treated HEK293T cells following the same procedure as described above. The nested PCR was performed using Q5 High-Fidelity polymerase (NEB) according to the manufacturer’s protocol. The first PCR amplicons were purified by gel extraction, and the second PCR amplicons were purified using Hieff NGS DNA selection beads (Yeasen). The concentration of the purified amplicons was determined using a NanoDrop spectrophotometer (Tiangen). Subsequently, the amplicons were pooled and subjected to NGS using an Illumina MiSeq platform, generating 300 bp paired-end reads.

The deep sequencing amplicon libraries were sequenced in PE150 mode. Raw paired-end reads were subjected to sublibrary barcode removal (first 6 bp) using Cutadapt (v1.15, available at https://github.com/marcelm/cutadapt). Subsequently, reads were aligned to their respective reference sequences using Bowtie 2 (v2.5.1) [27] with the following parameters: “--no-unal -N 1 -L 10 --no-mixed --very-sensitive”. Only one of the paired-end reads, positioned nearest to the gRNA target site, was retained for subsequent analysis. Editing events were quantified using CRISPResso2 (v2.2.12) [28], executed with the following parameters: “--exclude_bp_from_left 0 --exclude_bp_from_right 0 --quantification_window_center − 3 --ignore_substitutions”.

### Production of MC DNA

The production of MC DNA was carried out following a modified protocol based on a previous publication by Mark Kay [29]. Initially, parental plasmid (PP) DNAs listed in supplementary Table S1 were constructed and assembled in a vector (a gift from Penghui Zhou lab) using Gibson assembly. Subsequently, PP DNA was introduced into the ZYCY10P3S2T strain of *Escherichia coli.* The *Escherichia coli* containing PP DNA was cultured in Terrific Broth (pH 7.0) at 37 °C with shaking at 250 rpm until the OD600 reached 3–4. The MC DNA was then produced by mixing the *Escherichia coli* culture with an induction mix containing 20% arabinose and incubating at 30 °C for 7 h with shaking at 250 rpm. Finally, the MC DNA was isolated using the EndoFree Plasmid Kit (Tiangen) following the manufacturer’s protocol and verified by DNA gel electrophoresis.

### Immunofluorescence

HEK293T cells were seeded on glass coverslips and transfected with either TSP^scp^/PP-Cas9 or TSP^scp^/MC-Cas9 DNA. After 48 h of transfection, the cells were fixed with 4% paraformaldehyde for 20 min and then permeabilized with PBS containing 0.25% Triton X-100 for 30 min at room temperature. Following permeabilization, the cells were blocked in PBST (PBS with 0.1% Tween 20) containing 5% bovine serum albumin (BSA) at room temperature for 2 h and incubated with the primary anti-HA antibody (M180-3, MBL) overnight at 4 °C, followed by incubation with Alexa Fluor® 488-conjugated secondary antibody for 2 h. After staining with DAPI for 3 min and washing with PBST three times, the cells were mounted using a fluorescent mounting medium (P0131, Beyotime) and imaged using a confocal microscope.

### Evaluation of in vivo genome editing of CRISPR system delivered by TSP^scp^

MC DNAs expressing Cre-Cas9 fusion and MC sgRNA targeting *Il-4Ra* were mixed at weight ratio of 1:1, followed by complexing with TSP^scp^ (100 µg/µL) at weight ratio of 40:1. Complex of TSP (100 µg/µL) and the same MC Cre-Cas9/MC sgRNA was prepared at weight ratio of 20:1. 15 µL of each complex was intracutaneously injected into the dorsal skin of transgenic Ai14 mice (*n* = 3). 15 µL of PBS was injected as a control. Mice were sacrificed, and skin tissue at the injection spots were collected 21 days post-injection. The fresh skin tissue was dissociated into single cells using a combined enzymatic-mechanical tissue dissociation protocol involving dispase II. Tdtomato-positive cells were sorted by FACS on MoFlo XDP (Beckman). Genomic DNA was extracted, and genomic editing efficiency was analyzed using Tide-seq. The study was repeated three times independently with three mice in each group.

## Results and discussion

### Characterization of TSP^scp^/pDNA

**Fig. 2:**
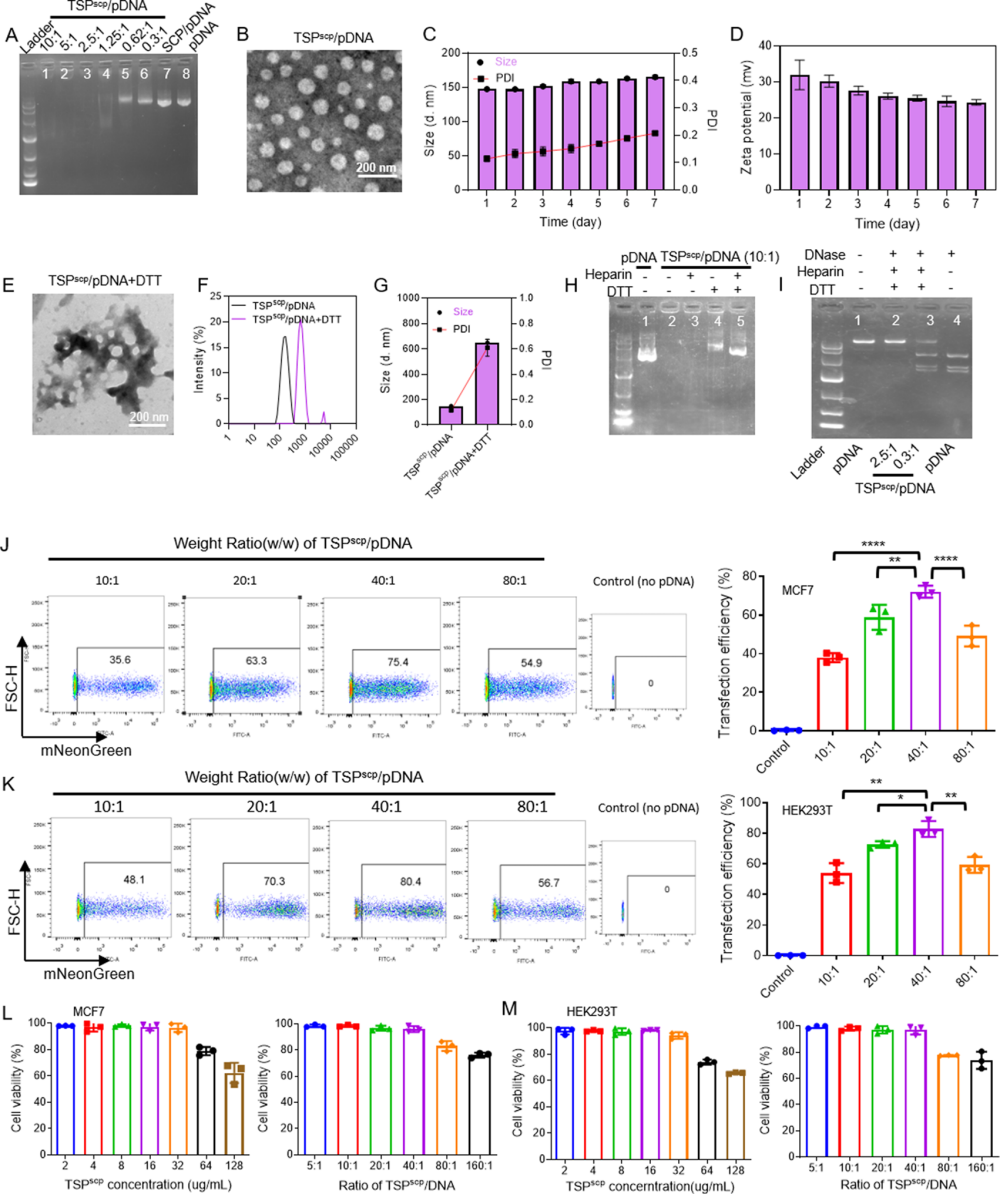
Characterization of TSP^scp^/pDNA complexes. (**A**) Binding ability of TSP^scp^ to pDNA. Lane 1–6, TSP^scp^/pDNA at different weight ratios. Lane 7, SCP/pDNA complex. Lane 8, pDNA only. (**B**) TEM image of TSP^scp^/pDNA particle at weight ratio of 10:1. (**C**) Particle size and PDI changes of TSP^scp^/pDNA after storage for 7 days. (**D**) Zeta potential changes of TSP^scp^/pDNA after storage for 7 days. (**E**) TEM image of TSP^scp^/pDNA after DTT treatment. (**F**) Size and (**G**) PDI changes of TSP^scp^/pDNA complexes with or without DTT treatment. (**H**) Release of pDNA from TSP^scp^/pDNA complexes with heparin and/or DTT treatment. (**I**) pDNA protected by TSP^scp^. Lane 1, pDNA only. Lane 2–3, TSP^scp^/pDNA complexes treated with DNase followed by heparin and DTT treatment. Lane 4, pDNA treated with DNase. (J&K) Transfection efficiency of TSP^scp^/pDNA on MCF7 (**J**) and HEK293T (**K**) quantified by flow cytometric analysis. pDNA (pmNG) expresses mNeoGreen fluorescent protein. Flow cytometry was carried out 48 h post transfection. (**L**&**M**) Cytotoxicity analysis of TSP^scp^ at different concentration and TSP^scp^/pDNA at different weight ratio in MCF7 (**L**) and HEK293T (**M**). Data represents as mean ± SD. *n* = 3 per group. ∗*P* < 0.05, ∗∗*P* < 0.01, ∗∗∗∗*P*< 0.0001. *P*-values are from ANNOVA analysis and Sidak’s multiple comparisons test

In this study, we expected that bioreducible TSP could enhance the transfection efficacy of CRISPR system, and the incorporation of cell-penetration peptide SCP could further improve the transfection by facilitating the cellular uptake of pDNA with minimal cytotoxicity. TSP was synthesized by conjugating both Tween 85 and PEI 2 K to pre-activated DTDP, then SCP was conjugated to PEI through amide reaction. ^1^H NMR confirmed the successful conjugation of SCP to TSP (Supplementary Figs. S2, S3). To evaluate the DNA binding capability of TSP^scp^, we performed a gel retardation assay for the complexes of TSP^scp^/pmNG prepared at various weight ratios ranging from 0.3:1 to 10:1. As illustrated in Fig. 2A, partial pDNA migration was observed at weight ratios of 0.3:1 to 1.25:1, while no pDNA migration was observed at weight ratios of 2.5:1 and above, suggesting that TSP^scp^ could completely condense pDNA when weight ratio is higher than 2.5:1. In contrast, the controls of SCP/pmNG at weight ratio of 10:1 and pmNG only exhibited almost no gel retardation, indicating that SCP was lack of the ability to compress pDNA. TSP^scp^/pDNA complex appeared as small spherical particles under TEM with a size of approximately 90 nm (Fig. 2B), and the DLS data showed that TSP^scp^/pmNG complex has a hydrodynamic particle size of approximately 150 nm with a polydispersity index (PDI) of around 0.11 and a zeta potential of approximately 32 mV. Since TEM indicates the size of dried particles, whereas DLS measures the hydrodynamic size, thus, the size measured by TEM is usually smaller than DLS. In addition, TSP^scp^/pDNA complex was relatively stable after storage at 4 °C for several days, as evidenced by the no obvious changes in size, PDI and zeta potential (Fig. 2C&D).

To assess the bioreducibility, TSP^scp^/pDNA complex was treated with DTT and/or heparin for 24 h and underwent the same characterization processes as described above. As shown in Fig. 2E, many TSP^scp^/pDNA particles treated with DTT appeared to be dissociated, leading to decreased zeta potential and increased size and PDI (Fig. 2F&G and Supplementary Fig. S4). The release of DNA from TSP^scp^/pDNA was also redox-responsive. As depicted in Fig. 2H, most of pDNA migrated in the agarose gel after incubation with DTT, possibly due to the redox-responsive degradation of TSP^scp^ that further lead to the effective release of the compressed pDNA. Furthermore, the addition of both heparin and DTT resulted in the complete release of pDNA, which could be attributed to the redox-responsive degradation of TSP^scp^ by DTT and the competitive binding of heparin with the PEI component. Interestingly, TSP^scp^/pDNA treated with heparin alone exhibited limited ability to release DNA, suggesting that there should be other interactions to help compress pDNA in addition to the electrostatic interaction.

We further investigated the protective capability of TSP^scp^ to the loaded pDNA from digestion. TSP^scp^/pDNA complexes at weight ratios of 0.3:1 and 2.5:1 were subjected to DNase treatment, followed by DTT and heparin treatment for pDNA release. As a control, the same amount of naked pDNA with or without DNase treatment was also prepared. The agarose gel analysis in Fig. 2I revealed that pDNA in TSP^scp^/pDNA complex at a weight ratio of 2.5:1 remained intact after treatment with DNase, while pDNA in TSP^scp^/pDNA complex at a weight ratio of 0.3:1 was partially digested by DNase, suggesting that TSP^scp^ could efficiently protect pDNA cargo from digestion, particularly at or potentially over the weight ratio of 2.5:1.

### TSP^scp^ promotes transfection by facilitating cellular uptake and lysosome escape

We first optimized the best weight ratio between TSP^scp^ and pmNG for transfection. TSP^scp^/pmNG complexes at different weight ratios (10:1, 20:1, 40:1, and 80:1) were incubated with MCF7 and HEK293T cell lines, and the green fluorescence from mNeonGreen (mNG) encoded by pmNG was then determined by flow cytometry. As shown in Fig. 2J&K, the transfection efficiency of TSP^scp^/pmNG was found to be highest at the weight ratio of 40:1 in both cell lines. Furthermore, the cytotoxicity of TSP^scp^ at different concentrations and TSP^scp^/pDNA complexes at various weight ratios were also evaluated by MTT method. As depicted in Fig. 2L&M, the viability of both cell lines slightly decreased to approximately 80% when the concentration of TSP^scp^ reached 64 µg/mL, indicating that TSP^scp^ is considered safe when used at concentrations lower than 64 µg/mL. In the case of TSP^scp^/pmNG complexes, no cytotoxicity was observed when the weight ratio was below 40:1, but slightly visible at a ratio of 80:1, which may explain the highest transfection efficiency observed at the ratio of 40:1 in Fig. 2J&K. Therefore, the weight ratio of 40:1 was selected for CRISPR transfection in the subsequent experiments.

**Fig. 3:**
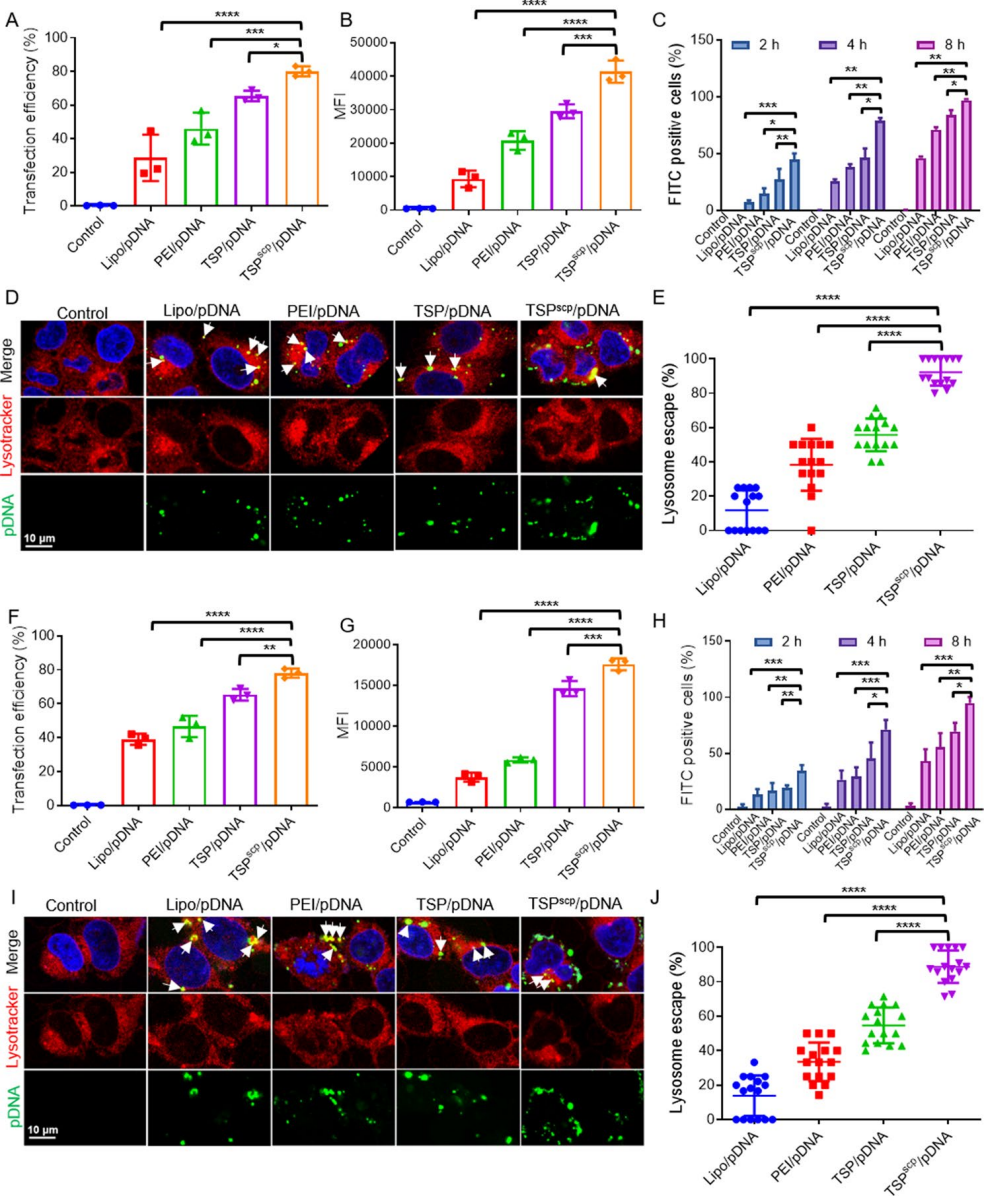
TSP^scp^ promotes delivery of CRISPR pDNA by facilitating cellular uptake and lysosome escape. (**A**) Transfection efficiency of TSP^scp^/pDNA, TSP/pDNA, PEI/pDNA and Lipo2000 (Lipo)/pDNA complexes in MCF7 cells. Flow cytometric analysis was performed 48 h post transfection. Plasmid pCas12f expressing mCherry tagged dCas12f-VPR was used. (**B**) Mean fluorescence intensity (MFI) of mCherry-dCas12f from flow cytometric analysis. (**C**) Cellular uptake of TSP^scp^/pDNA, TSP/pDNA, PEI/pDNA and Lipo/pDNA complexes in MCF7. pCas12f DNA was labelled with FITC. Flow cytometry was performed 2 h, 4 h, and 8 h post transfection. (**D**) Colocalization of TSP^scp^/pDNA, TSP/pDNA, PEI/pDNA and Lipo/pDNA complexes with lysosome in MCF7. pDNA was labelled with FITC. Lysosome staining with red lysotracker was performed 8 h after transfection, followed by confocal imaging. Red, LysoTracker. Green, FITC-pDNA. Blue, DAPI stained nuclei. The white arrows point to non-escaped pDNA that overlapped with Lysosome. Scale bar, 10 μm. (**E**) Percentages of FITC-pDNA escaped from lysosome quantified from the confocal images. Each dot represents a field of view. (**F-J**) Transfection efficiency, cellular uptake, and lysosome colocalization were similarly assayed in HEK293T. Data in **A-C** & **F-H** panels represent as mean ± SD, *n* = 3 per group. ∗*P* < 0.05, ∗∗*P* < 0.01, ∗∗∗*P* < 0.001, ∗∗∗∗*P* < 0.0001. *P*-values are from ANNOVA analysis and Sidak’s multiple comparisons test

We then compared the ability of TSP^scp^ to promote transfection with other commercial transfection agents (Lipo2000 and PEI 25 K, identified as Lipo and PEI, respectively, for short) at their optimal transfection conditions. Vectors were complexed with pCas12f that expresses mCherry tagged dCas12f nuclease, and the complexes were incubated with MCF7 or HEK293T cells for 48 h. The flow cytometry analysis depicted in Fig. 3A&F showed that transfection efficiency of pCas12f complexed with TSP^scp^ was as high as 80%, which was significantly higher than that complexed with TSP, PEI, and Lipo. The mean fluorescence intensity (MFI) of mCherry-dCas12f carried by TSP^scp^ was also significantly increased, compared to other groups (Fig. 3B&G). Collectively, these findings suggest that SCP conjugation to TSP can greatly improve the transfection of CRISPR pDNA.

To Figure out the mechanism that TSP^scp^ enhanced transfection, we tested cellular uptake efficiency of TSP^scp^/pCas12f. Complexes of FITC-labeled pCas12f with TSP^scp^, TSP, PEI 25 K, and Lipo were prepared, and then incubated with cells for 2 h, 4 h, or 8 h. As shown in Fig. 3C&H, the percentages of FITC-positive cells in TSP^scp^/pCas12f treated groups were all significantly increased compared to other groups at all time points. The higher cellular uptake of TSP^scp^/pCas12f than TSP/pCas12f indicated that the incorporation of SCP is beneficial for the internalization. Furthermore, the colocalization of TSP^scp^/pCas12f with lysosomes was also detected by staining with lysotracker red 8 h post-incubation to determine the lysosome escape ability. The confocal images showed that most of pCas12f (green) was non-overlapped with lysosomes (red) in the TSP^scp^/pCas12f transfection group compared to other groups (Fig. 3D&I), demonstrating that most pCas12f escaped from lysosomes. Higer cellular uptake and more lysosomes escape would be attributed to the higher transfection. These data all suggested that TSP^scp^ could improve cellular uptake and lysosomal escape efficiency, finally resulting in the enhanced transfection of CRISPR pDNA.

Safety is a fundamental requirement for the application of gene delivery systems. The cytotoxicity of TSP^scp^/pDNA complex in both HEK293T and MCF7 cells was compared with other complexes by MTT method. As excepted, both Lipo/pDNA and PEI/pDNA complexes cause toxicity to cells, reducing the cell viability to ~ 70% and 65%, respectively. On the contrary, TSP^scp^/pDNA and TSP/pDNA complexes showed no effect on cell viability, compared to control group (Supplementary Fig.S5 A & B), which suggested that TSP^scp^ is a potentially safe carrier for CRISPR system.

### TSP^scp^ promotes gene activation activity of CRISPR system

**Fig. 4:**
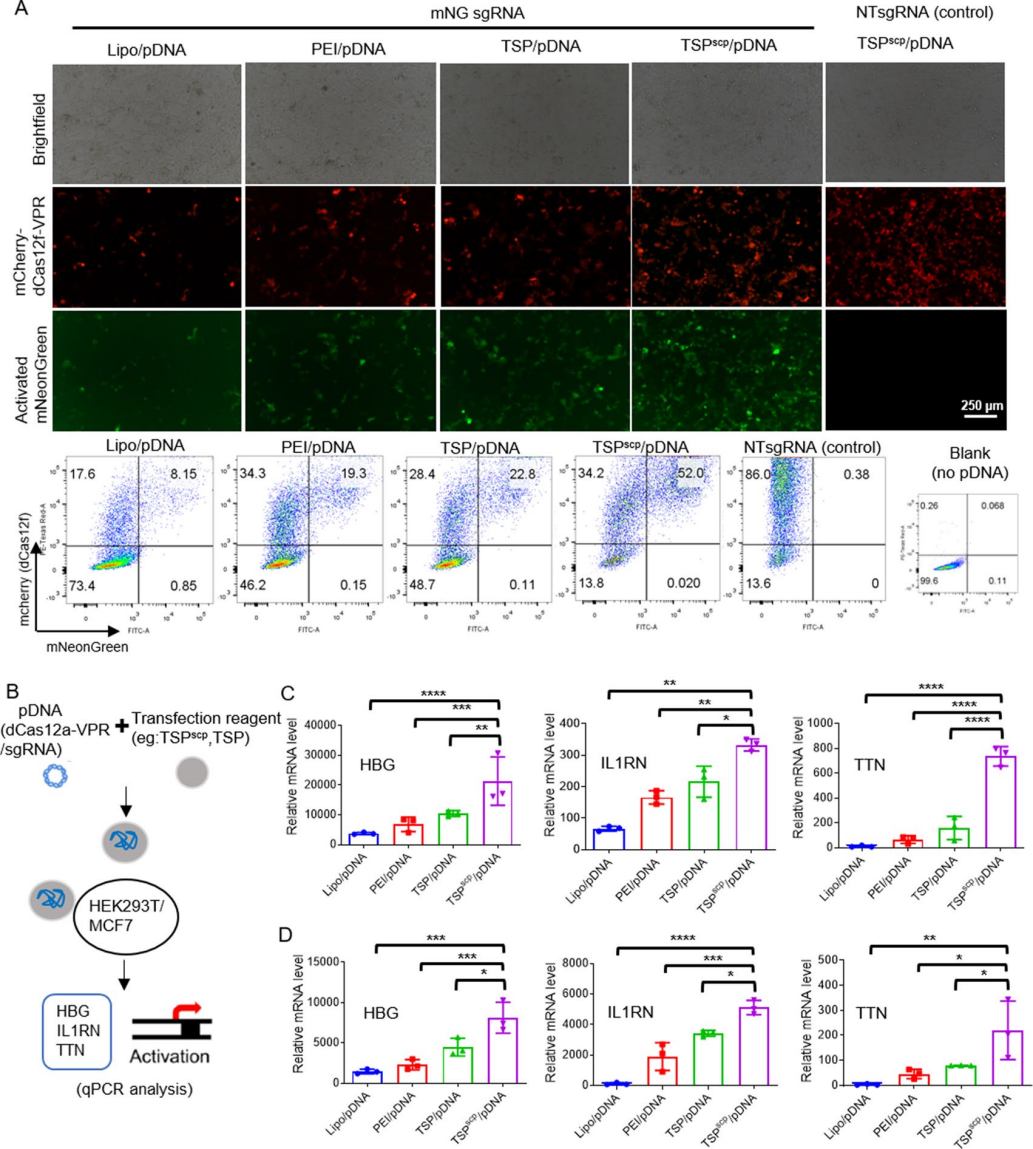
TSP^scp^ promotes gene activation activity of CRISPR-dCas system. (**A**) Activation of mNG expression by mCherry-dCas12f-VPR/sgRNA targeting mNG promoter in the HEK293T reporter cells after TSP^scp^/pDNA, TSP/pDNA, PEI/pDNA and Lipo/pDNA transfection. In the non-targeted (NT) sgRNA control, cells transfected with a non-targeted sgRNA. Fluorescence imaging and flow cytometry analysis were performed 48 h post transfection. Red, mCherry-dCas12f-VPR. Green, mNG. VPR (VP64-p65-Rta), a transcriptional activator. Scale bar, 250 μm. (**B**) Schematic analysis of endogenous gene activation by CRISPR-dCas12a delivered by TSP^scp^ and other transfection reagents. (**C-D**) qPCR analysis of targeted genes including HBG, IL1RN and TTN, in TSP^scp^/pDNA, TSP/pDNA, PEI/pDNA and Lipo/pDNA transfected cells. RNA extraction was performed 48 h post transfection. *n* = 3 per group. Data represents as mean ± SD, ∗*P* < 0.05, ∗∗*P* < 0.01, ∗∗∗*P*< 0.001, ∗∗∗∗*P* < 0.0001. *P*-values are from ANNOVA analysis and Sidak’s multiple comparisons test

Dead Cas (dCas) variant that lacks DNA cleavage activity can be fused with a transcription activation domain like VPR to activate gene expression by targeting the promoter region [30–32]. Since our data above has demonstrated that TSP^scp^ could enhance delivery of pCas12f expressing mcherry-dCas12f-VPR, we next tested whether TSP^scp^-mediated delivery could improve its gene activation activity by transfecting pDNAs expressing mCherry-dCas12f-VPR and sgRNA targeting mNG promoter in the HEK293T reporter cell line, followed by comparison of mNG expression that is activated by mCherry-dCas12f-VPR in different transfection conditions. Both the fluorescence images and flow cytometry analysis again revealed that percentage of the cell population expressing mCherry-dCas12f-VPR (red) is the highest (~ 86%) in TSP^scp^/pDNA among all groups. More importantly, we observed that the cell population with expression of mNG reporter (green) activated by dCas12f-VPR in TSP^scp^/pDNA group is approximately 52%, which is 6.4-, 2.7-, and 2.3-times higher than Lipo/pDNA, PEI/pDNA, and TSP/pDNA groups, respectively (Fig. 4A). By contrast, nearly no mNG expression was observed in the control group of TSP^scp^/pDNA, in which the pDNA expresses dCas12f-VPR/non-target (NT) sgRNA. Furthermore, we expanded our investigation to examine the influence of TSP^scp^ on gene activation by transfecting dCas12a-VPR/sgRNAs targeting three endogenous genes including HBG, IL1RN, and TTN in HEK293T and MCF7 cells (Fig. 4B). The qPCR analysis result showed that expression level of each target gene was significantly elevated in the TSP^scp^/pDNA group compared to others (Fig. 4C-D). All these findings suggest that TSP^scp^-mediated delivery of CRISPR pDNA could result in enhanced gene activation activity.

### TSP^scp^ enhances genomic editing activity of CRISPR system

**Fig. 5:**
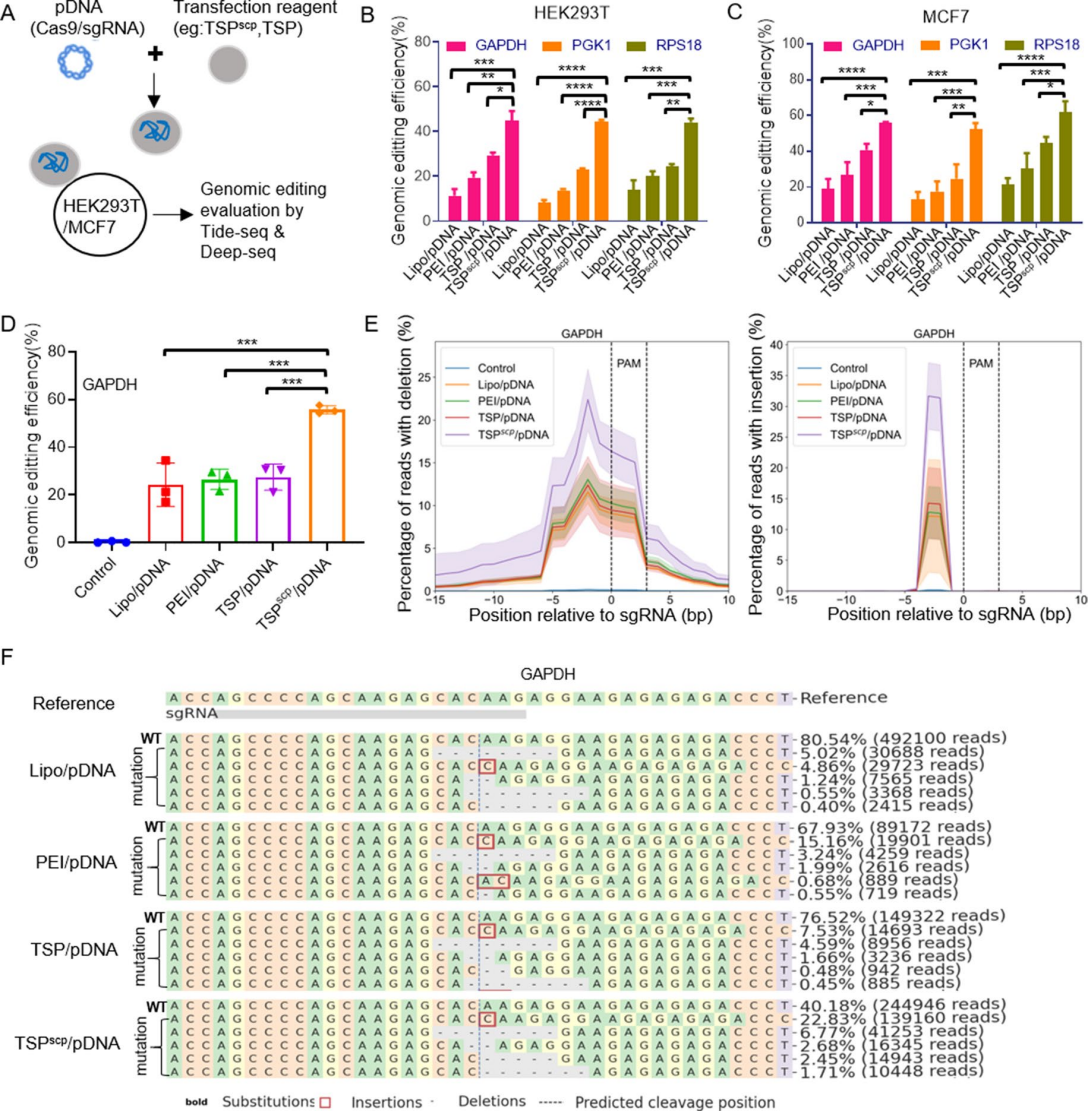
TSP^scp^ promotes genomic editing activity of CRISPR-Cas9. (**A**) Schematic experimental design of genomic editing evaluation by Tide-seq and Deep-seq after transfection of CRISPR pDNA via TSP^scp^ and other reagents. (**B, C**) Genomic editing efficiency analysis by Tide-seq after transfection of CRISPR-cas9/sgRNA targeting indicated genes in HEK293T and MCF7 cells respectively. (**D, F**) Genomic editing analysis of GAPDH by Deep-seq after transfection in HEK293T. Comparison of genomic editing efficiency (**D**), percentages of reads with deletion or insertion at indicated position (**F**), and top 5 indel mutations (**F**) among different transfection conditions. (**F**) Bold indicates substitution mutations. Red box indicates insertion mutation. Short dash indicates deletions. Data in **B-D** panels represents as mean ± SD, *n* = 3 per group. ∗ *P* < 0.05, ∗∗ *P* < 0.01, ∗∗∗*P* < 0.001, ∗∗∗∗ *P* < 0.0001. P values are from ANNOVA analysis and Sidak’s multiple comparisons test. The assay of each gene was repeated in 3 independent experiments

We next tested whether TSP^scp^-mediated delivery of CRISPR pDNA influence its genomic editing activity by transfecting pDNAs expressing Cas9/sgRNAs targeting GAPDH, PGK1, and RPS18 genes in HEK293T and MCF7 cells (Fig. 5A). As a simple assay that accurately determines the indel frequency of targeted genes induced by the CRISPR/Cas9 system [33], TIDE-seq was performed by sequencing PCR amplicons of each target gene from genomic DNA extracted 48 h post transfection. The results showed that for each target gene, genome editing efficiency in TSP^scp^/pDNA group was significantly increased than in other groups (Fig. 5B&C). To take GAPDH in HEK293T as an example, the average of genomic editing efficiency in TSP^scp^/pDNA, TSP/pDNA, PEI/pDNA and Lipo/pDNA groups were 44.7%, 29.3%, 19.3% and 11.3% respectively. Additionally, deep-seq was performed for more detailed base editing comparisons among different transfection groups in HEK293T. Likewise, the genomic editing efficiency of GAPDH (Fig. 5D), PGK1 and RPS18 (Supplemental Fig. S6A) was significantly elevated in TSP^scp^/pDNA compared to other groups. Moreover, for GAPDH, the percentage peak of deletion or insertion reads within 5 bp upstream of the PAM site in TSP^scp^/pDNA group was about 1.8 or 2 times higher than that of other groups (Fig. 5E), and the frequency of top 5 indel mutations were elevated up to 3 times higher in TSP^scp^/pDNA group than other groups (Fig. 5F). Similar results were also observed for PGK1 and RPS18 genes (Supplementary Fig. S6B&C). These results collectively suggest that TSP^scp^-mediated delivery enhances genomic editing activity of CRISPR system.

### TSP^scp^ further enhances genome editing activity of compact CRISPR pDNA

**Fig. 6:**
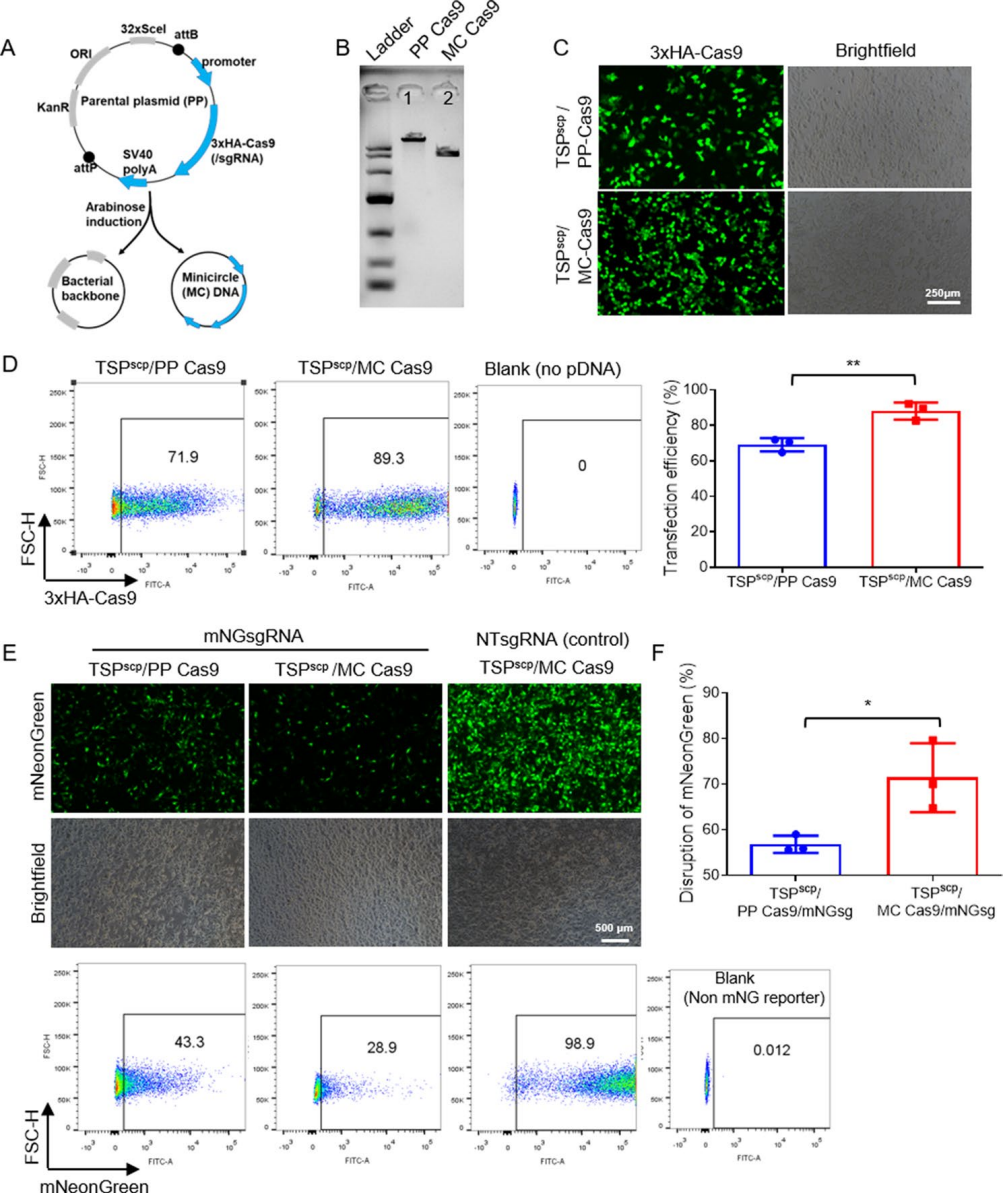
TSP^scp^ further enhances genome editing efficiency of condensed CRISPR system. (**A**) Schematic design of MC DNA produced from PP DNA after arabinose induction. (**A**) Gel electrophoresis of PP Cas9 and MC Cas9 DNAs. (**A**) Immunofluorescent images of HEK293T cells expressing 3xHA-Cas9 in TSP^scp^/PP-Cas9 and TSP^scp^/MC-Cas9 transfections. Immunostaining was performed with antibody against HA tag 48 h post transfection. (**A**) Transfection efficiency of TSP^scp^/PP-Cas9 vs. TSP^scp^/MC-Cas9, analyzed by flow cytometry after HA staining 48 h post transfection. No pDNA transfection was used as blank control. (**E**) Fluorescence images and flow cytometric analyses of cells expressing mNG reporter in TSP^scp^/PP Cas9/mNGsg and TSP^scp^/MCCas9/mNGsg transfections in HEK293T mNG reporter cell line. Non-target sgRNA control, TSP^scp^/MC-Cas9/NTsg transfection. Regular HEK293T cells without mNG reporter expression was used as a blank for gating in flow cytometry. (**F**) Percentage of cells with disrupted expression of mNG to evaluate genome editing efficiency. The disruption efficiency of each group was calculated by subtracting the percentage of mNG positive cells from 100. Mean values are presented with ± SD, *n* = 3 independent experiments. ∗*P* < 0.05, ∗∗*P* < 0.01, ∗∗∗*P* < 0.001, ∗∗∗∗*P* < 0.0001. *P*-values are from TTEST analysis

MC DNA, a condensed form of standard plasmid DNA devoid of the bacterial backbone, has been documented to boost cellular uptake and gene expression [34, 35]. We next tested whether combination of MC DNA of CRISPR system with TSP^scp^ could further enhance genome editing activity. To begin with, MC Cas9 was produced from the parental plasmid (PP) DNA expressing 3xHA tagged Cas9 following the procedure as indicated in Fig. 6A. As predicted, DNA size of MC Cas9 (lane 2) is about 3000 bp smaller than PP Cas9 (lane 1) as shown in gel electrophoresis (Fig. 6B). After transfections of MC-Cas9 and PP-Cas9 by TSP^scp^ in HEK293T cell, immunofluorescence images and flow cytometry were performed to compare expression difference of HA-Cas9. The results demonstrated that percentage of the cell population expressing 3xHA-Cas9 in TSP^scp^/MC DNA transfection group was up to 89%, which was significantly increased than in TSP^scp^/PP DNA group (about 69% in average) (Fig. 6C&D). Subsequently, transfections of MC Cas9/mNGsgRNA and PP Cas9/mNGsgRNA by TSP^scp^ in HEK293T-mNG reporter cells were carried out followed by fluorescence imaging and flow cytometry to evaluate genome editing efficacy. In the result, we observed more reduced expression of mNeonGreen in TSP^scp^/MC than in TSP^scp^/PP group in fluorescence images (Fig. 6E). Moreover, flow cytometry result showed that percentage of cells expressing mNeonGreen is 28.9% in TSP^scp^/MC and 43.3% in TSP^scp^/PP group, suggesting more efficient disruption of mNeonGreen expression resulted from genome editing in TSP^scp^/MC (Fig. 6E). As expected, percentage of cells with disrupted mNG expression is significantly higher in TSP^scp^/MC than in TSP^scp^/PP group (Fig. 6F), indicating enhanced genome editing in TSP^scp^/MC group. By contrast, percentage of mNG positive cells is about 99% in the NTsgRNA control (Fig. 6E). These findings suggest that TSP^scp^ in combination with MC DNA can further promote genome editing efficiency of CRISPR, which holds TSP^scp^/MC DNA great potential for CRISPR-based genome editing in vivo.

### TSP^scp^ facilitates delivery and activity of CRISPR-Cas9 in vivo

**Fig. 7:**
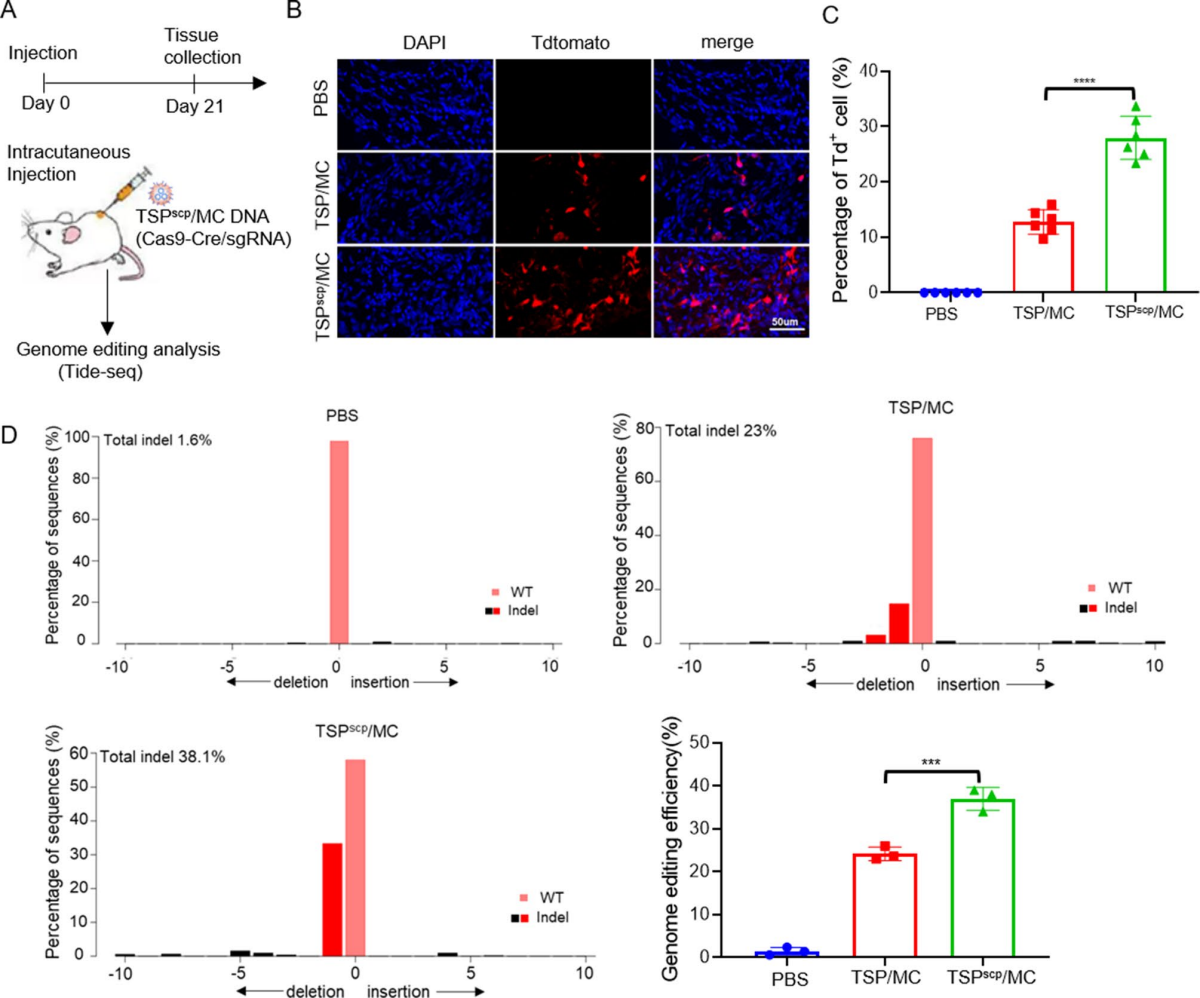
TSP^scp^-mediated delivery of MC CRISPR enhances genome editing in vivo. (**A**) Schematic of the animal study. (**B**) Fluorescence image of stained skin tissue from Ai14D mice after injection of TSP/MC Cas9-Cre/Il4-Ra sgRNA, TSP^scp^/MC Cas9-Cre/Il4-Ra sgRNA or PBS. Blue, DAPI stained nuclei. Red, Tdtomato reporter. (**C**) Quantification of tdTomato positive cells in fluorescent images of skin tissue. Each dot represents a field of view. (**D**) Genome editing efficiency analysis by Tide-seq. Mean values are presented with ± SD. ∗∗∗*P* < 0.001. ∗∗∗∗*P* < 0.0001. P values are from ANNOVA analysis and Sidak’s multiple comparisons test. The assay was repeated in three independent experiments with three mice in each group

To test whether TSP^scp^ facilitates genome editing efficacy of CRISPR in vivo, complexes of MC DNA expressing Cas9-Cre/sgRNA targeting *Il-4Ra* gene with TSP^scp^ or TSP alone were injected intracutaneously into transgenic Ai14D mice, followed by tissue collection and genome editing analysis via Tide-seq 21 days post injection (Fig. 7A). The transgenic Ai14D mice contains loxP-flanked STOP cassette that inhibits transcription of the tdTomato reporter and expression of Cre recombinase can activate transcription of tdTomato by binding to loxP sequence, thus tdTomato expression was detected in skin tissue by fluorescence imaging after injection to evaluate whether Cas9-Cre/sgRNA was delivered successfully. In the result, significantly more tdTomato-positive (td^+^) cells (red) were observed in TSP^scp^/MC (about 28%) compared to TSP/MC group (approximately 12%) in fluorescence images (Fig. 7B&C), suggesting more efficient delivery of MC Cas9-Cre by TSP^scp^. On the contrary, no tdTomato expression was detected in the PBS injection control (Fig. 7B). Subsequently, genome editing efficacy in td^+^ cells was evaluated by Tide-seq. As a result, genome editing frequency in TSP^scp^/MC group was approximately 37%, which was significantly higher than the TSP/MC group (approximately 24%) (Fig. 7D). In summary, TSP^scp^ enhances the in vivo delivery of CRISPR system, resulting in improved genome editing activity. Considering that *Il-4Ra* inhibitors have been used in clinical Atopic Dermatitis treatment, TSP^scp^/MC DNA of the CRISPR system could be potentially applied as an alternative therapeutic approach.

Our data indicate that the novel polymer TSP^scp^ has the potential to serve as a carrier for CRISPR-based gene therapy due to its advantages, such as cost-effective preparation, minimal toxicity, high loading capacity, and absence of immunity risks compared to other reported vectors like adeno-associated viral vectors [36] or lipid nanoparticle [15] for CRISPR system delivery. However, further studies need to be done to investigate the detailed mechanism that TSP^scp^ enhance transfection efficacy, which will be helpful for further optimization and final in vivo CRISPR delivery. Moreover, the specificity of TSP^scp^ towards specific cell types remains uncertain, warranting additional experiments across a diverse array of cell types.

## Conclusions

In conclusion, this study highlights the effectiveness of a cell-penetrating peptide-conjugated bioreducible polymer, TSP^scp^, in combination with size-compacted MC DNA as a versatile system for efficient CRISPR delivery. In vitro experiments conducted on HEK293T and MCF7 cell lines demonstrated that TSP^scp^-mediated delivery of CRISPR systems surpassed commercial transfection reagents, enhancing gene activation and genome editing by approximately two to four times. Moreover, the use of MC DNA, with a more condensed size, improved transfection efficiency compared to PP DNA delivered by TSP^scp^, leading to further enhancements in genome editing efficiency. Importantly, our in vivo investigation revealed that the novel carrier TSP^scp^/MC DNA enhances the delivery efficiency of the CRISPR system in skin tissue, resulting in significantly higher genome editing efficiency compared to TSP/MC DNA. Future research endeavors will focus on evaluating the application of TSP^scp^/MC DNA-mediated CRISPR system delivery in treating skin diseases like Atopic Dermatitis. In essence, this study introduces a novel and efficient delivery tool, TSP^scp^/MC DNA, for CRISPR systems, offering great promise for the advancement of CRISPR-based gene therapy applications through enhanced genome editing capabilities.

### Electronic supplementary material

Below is the link to the electronic supplementary material.

Supplementary Material 1

## Data Availability

No datasets were generated or analysed during the current study.
