# Supplementary material for: Short cell-penetration peptide conjugated bioreducible polymer enhances gene editing of CRISPR system
Source: J Nanobiotechnology. 2024 May 24;22:284. doi: 10.1186/s12951-024-02554-w (PMC11127455; doi:10.1186/s12951-024-02554-w)
Supplement: Supplementary file 1 — Supplementary Material 1 [file 12951_2024_2554_MOESM1_ESM.docx]

**Supplementary data and material**


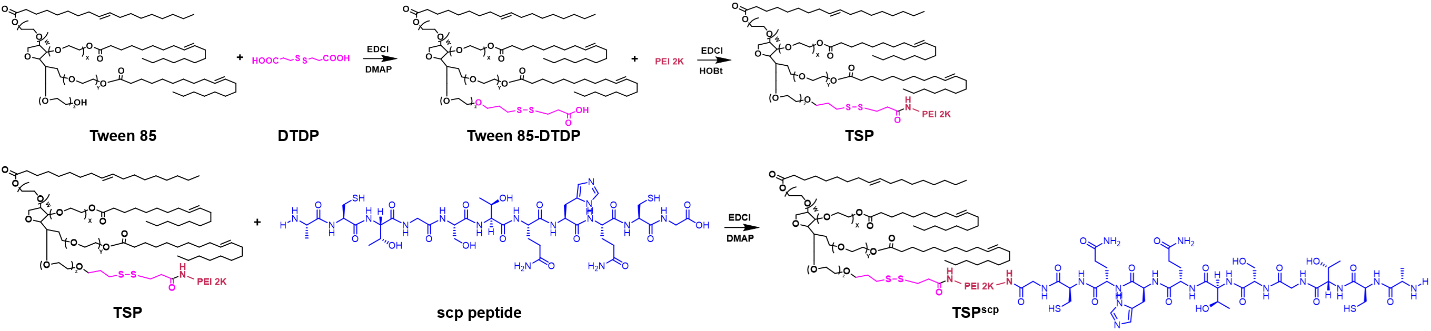


Supplementary Figure 1. Systhesis of TSP^scp^.


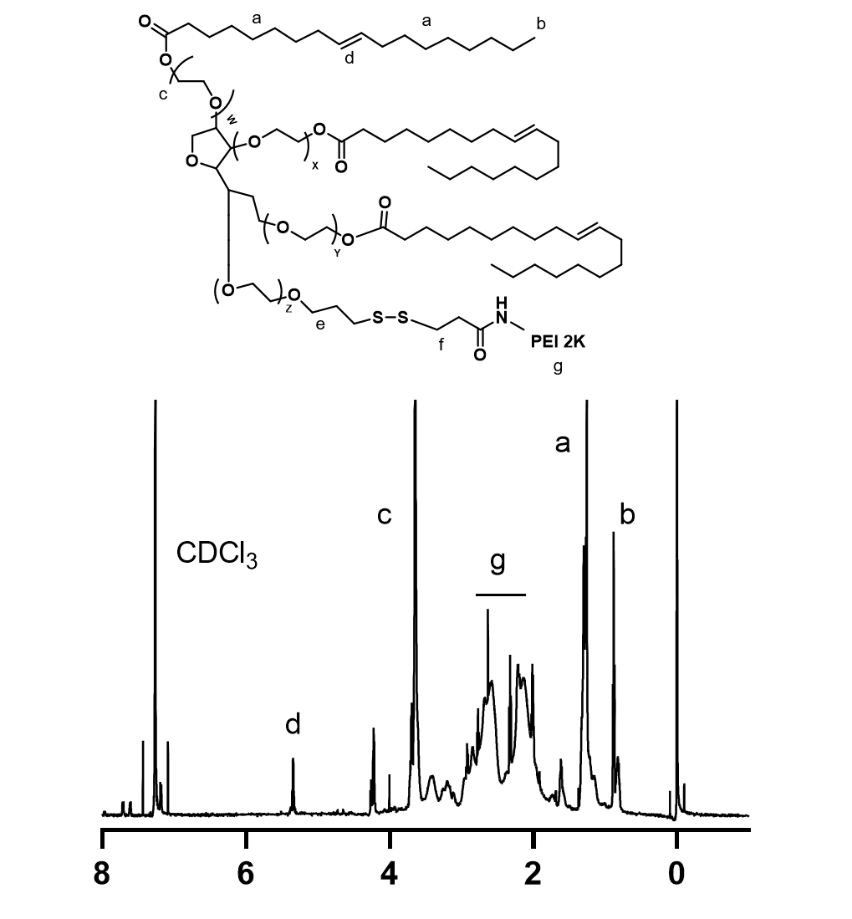


Supplementary Figure 2. ^1^HNMR of TSP (CDCl_3_): 5.34 (m, 6H), 3.65 (t, 80H), 1.27 (m, 60H), 0.88 (t, 9H).


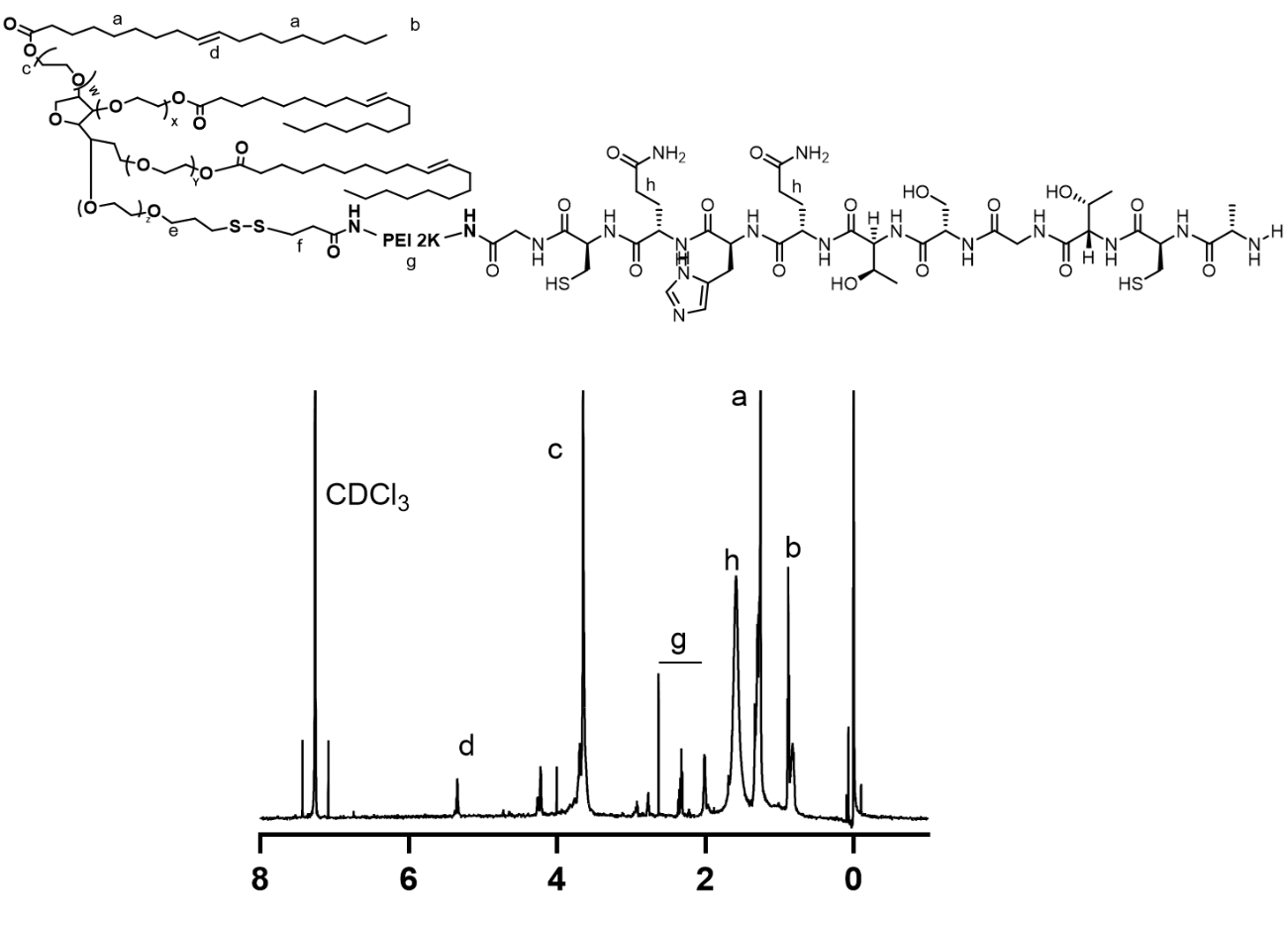


Supplementary Figure 3. ^1^HNMR of TSP^scp^ (CDCl_3_): 7.37 (d, 1H), 6.79 (d, 1H), 5.34 (m, 6H), 4.26 (m, 3H), 4.22 (m, 8H), 3.69 (m, 9H), 3.65 (t, 80H), 2.70-2.95 (m, 4H), 1.30 (d, 3H), 1.25 (m, 60H), 0.88 (t, 9H), 0.82 (t, 6H).

Supplementary Figure 4. Zeta potential comparison of TSP^scp^/pDNA before and after dissociation.


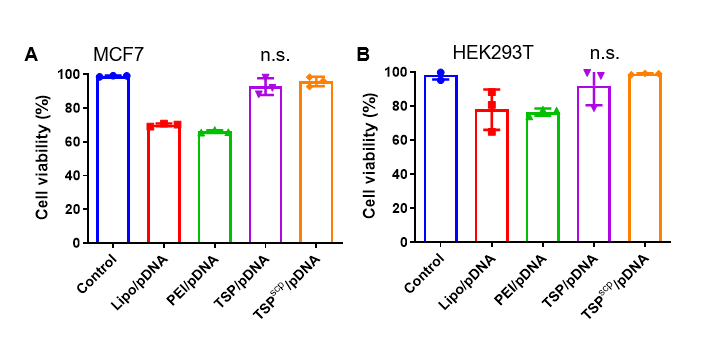
Supplementary Figure 5. Cytotoxity of TSP^scp^/pDNA transfected cells.


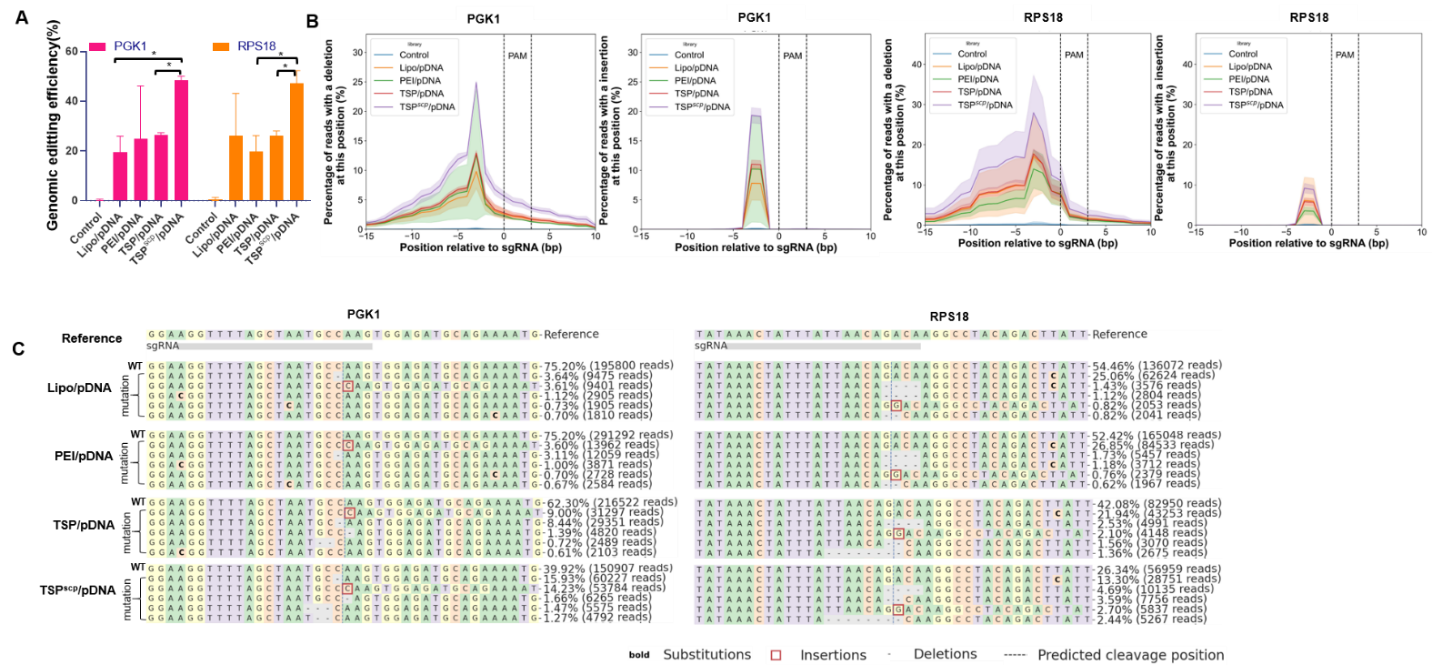


Supplementary Figure 6. Frequencies of top 5 indel mutations resulted from genome editing in PGK1 and RPS18 genes by CRISPR-Cas9. (A) Genomic editing efficiency of different complexes on PGK1 and RPS18. (B) Percentages of reads with deletion or insertion at indicated position after transfection with different complexes. (C) Top 5 indel mutations post transfection with different complexes.

Table S1. Plasmids used in this study.

| **Plasmid** | **Inserted coding gene** | **Function** | **Figure** |
| --- | --- | --- | --- |
| pmNG | mNeonGreen | N/A | Figure2 |
| pCas12a | mcherry-dLbCas12a-VPR | transcriptional activation | Figure4 |
| pCas12f | dUn1Cas12f-VPR | transcriptional activation | Figure3-4 |
| pCas9 | mcherry-SpCas9 | genome editing | Figure5 |
| PP Cas9 | 3xHA-SpCas9 | genome editing | Figure6 |
| MC Cas9 | 3xHA-SpCas9 | genome editing | Figure6 |
| PP mNGsgRNA | sgRNA targeting mNeonGreen | genome editing | Figure6 |
| MC mNGsgRNA | sgRNA targeting mNeonGreen | genome editing | Figure6 |
| PP Cas9-Cre | SpCas9-NLS-Cre | genome editing | Figure7 |
| MC Cas9-Cre | SpCas9-NLS-Cre | genome editing | Figure7 |
| PP *Il-4Ra*sgRNA | sgRNA targeting *Il-4Ra* | genome editing | Figure7 |
| MC *Il-4Ra*sgRNA | sgRNA targeting *Il-4Ra* | genome editing | Figure7 |

Table S2. sgRNA sequences used in this study.

| **CRISPR/Cas** | **Gene name** | **sgRNA sequence** | **PAM** | **CRISPR activity** | **Figure** |
| --- | --- | --- | --- | --- | --- |
| dUn1Cas12f-VPR | *mNG* | CGTGTCACTCGTGATCCAGTAGC | TTTA | transcriptional activation | Figure4 |
| dLbCas12a-VPR | *HBG* | AACTACAGGCCTCACTGGAGCTA | TTTA | transcriptional activation | Figure4 |
|  | *IL1RN* | CAGGAGGGTGACTCAGGCTAGCA | TTTC | transcriptional activation | Figure4 |
|  | *TTN* | TGGGGGAAGGGAACAGGACTGGG | TTTA | transcriptional activation | Figure4 |
| SpCas9 | *GAPDH* | AGCCCCAGCAAGAGCACAAG | AGG | genome editing | Figure5 |
|  | *PGK1* | AGGTTTTAGCTAATGCCAAG | TGG | genome editing | Figure5 |
|  | *RPS18* | AAACTATTTATTAACAGACA | AGG | genome editing | Figure5 |
|  | *mNG* | CAACCCCAACGACGGCTACG | AGG | genome editing | Figure6 |
|  | *Il-4Ra* | GAAGAAGACAGACTTCCCGA | AGG | genome editing | Figure7 |

Table S3. Primers used in this study.

| **Name** | **Sequence (5’-3’)** |
| --- | --- |
| **qPCR primers** | |
| GAPDH-qF | AGAAGGCTGGGGCTCATTTG |
| GAPDH-qR | AGGGGCCATCCACAGTCTTC |
| HBG-qF | GCTGAGTGAACTGCACTGTGA |
| HBG-qR | GAATTCTTTGCCGAAATGGA |
| IL1RN-qF | GGAATCCATGGAGGGAAGAT |
| IL1RN-qR | TGTTCTCGCTCAGGTCAGTG |
| TTN-qF | TGTTGCCACTGGTGCTAAAG |
| TTN-qR | ACAGCAGTCTTCTCCGCTTC |
| **primers for amplicon before TIDE-seq** | |
| GADPH-flank. F | CACATGGCCTCCAAGGAGTAA |
| GADPH-flank. R | GCCCCAGACCCTAGAATAAGAC |
| RPS18-flank. F | GCCTACCTCGACTCAGCATT |
| RPS18-flank. R | GTGTGGGCAACTGAGTAGGG |
| PGK1-flank. F | AAGTGGGGGAAATCTGGCTT |
| PGK1-flank. R | GGGTGCAGTGAAGATGAGCT |
| *Il-4Ra*-flank. F | tcagctttacctctgcaggc |
| *Il-4Ra*-flank. R | ccgctgttctcaggtgacat |
| **Deep-seq library amplicon primers** | |
| PGK1.R1.1F | AcgCTCTTTCCCTACACGACGCTCTTCCGATCTAGTCAAagagctggcatgttattggga |
| PGK1.R1.1R | ctACTGGAGTTCAGACGTGTGCTCTTCCGATCTAGTTCCccagggtgcagtgaagatga |
| GAPDH.R1.1F | acgCTCTTTCCCTACACGACGCTCTTCCGATCTAGTCAACACATGGCCTCCAAGGAGTAA |
| GAPDH.R1.1R | ctACTGGAGTTCAGACGTGTGCTCTTCCGATCTAGTTCCGCCCCAGACCCTAGAATAAGAC |
| RPS18.R1.1F | acgCTCTTTCCCTACACGACGCTCTTCCGATCTAGTCAAcgctcttctttttccccaacc |
| RPS18.R1.1R | ctACTGGAGTTCAGACGTGTGCTCTTCCGATCTAGTTCCacccagcactttaacattccc |
| P5 | AATGATACGGCGACCACCGAGATCTACACCGTGATACACTCTTTCCCTACACGACGC |
| P7 | CAAGCAGAAGACGGCATACGAGATCATGCCTAGTGACTGGAGTTCAGACGTGTGCT |

**DNA sequences:**

**---dUn1Cas12f-Nucleoplamin NLS-VPR**

ggtatccacggagtcccagcagccatggccaagaacacaattacaaagacactgaagctgaggatcgtgagaccatacaacagcgctgaggtcgagaagattgtggctgatgaaaagaacaacagggaaaagatcgccctcgagaagaacaaggataaggtgaaggaggcctgctctaagcacctgaaagtggccgcctactgcaccacacaggtggagaggaacgcctgtctgttttgtaaagctcggaagctggatgataagttttaccagaagctgcggggccagttccccgatgccgtcttttggcaggagattagcgagatcttcagacagctgcagaagcaggccgccgagatctacaaccagagcctgatcgagctctactacgagatcttcatcaagggcaagggcattgccaacgcctcctccgtggagcactacctgagcagagtgtgctacagaagagccgccgagctctttaagaacgccgctatcgcttccgggctgaggagcaagattaagagtaacttccggctcaaggagctgaagaacatgaagagcggcctgcccactacaaagagcgacaacttcccaattccactggtgaagcagaaggggggccagtacacagggttcgagatttccaaccacaacagcgactttattattaagatcccctttggcaggtggcaggtcaagaaggagattgacaagtacaggccctgggagaagtttgatttcgagcaggtgcagaagagccccaagcctatttccctgctgctgtccacacagcggcggaagaggaacaaggggtggtctaaggatgaggggaccgaggccgagattaagaaagtgatgaacggcgactaccagacaagctacatcgaggtcaagcggggcagtaagatttgcgagaagagcgcctggatgctgaacctgagcattgacgtgccaaagattgataagggcgtggaccccagcatcatcggagggatcgccgtgggggtcagaagccccctcgtgtgcgccatcaacaacgccttcagcaggtacagcatctccgataacgacctgttccactttaacaagaagatgttcgcccggcggaggattttgctcaagaagaaccggcacaagcgggccggacacggggccaagaacaagctcaagcccatcactatcctgaccgagaagagcgagaggttcaggaagaagctcatcgagagatgggcctgcgagatcgccgatttctttattaagaacaaggtcggaacagtgcagatggagaacctcgagagcatgaagaggaaggaggattcctacttcaacattcggctgagggggttctggccctacgctgagatgcagaacaagattgagtttaagctgaagcagtacgggattgagatccggaaggtggcccccaacaacaccagcaagacctgcagcaagtgcgggcacctcaacaactacttcaacttcgagtaccggaagaagaacaagttcccacacttcaagtgcgagaagtgcaactttaaggagaacgccgcctacaacgccgccctgaacatcagcaaccctaagctgaagagcactaaggagagacccaaaaggccggcggccacgaaaaaggccggccaggcaaaaaagaaaaagggatgctttagacgattttgacttagatatgcttggttcagacgcgttagacgacttcgacctagacatgttaggctcagatgcattggacgacttcgatttagatatgttgggctccgatgccctagatgactttgatctagatatgctaggaggaagcggaggaggaggtagcggacctaagaaaaagaggaaggtggcggccgctggatccccttcagggcagatcagcaaccaggccctggctctggcccctagctccgctccagtgctggcccagactatggtgccctctagtgctatggtgcctctggcccagccacctgctccagcccctgtgctgaccccaggaccaccccagtcactgagcgctccagtgcccaagtctacacaggccggcgaggggactctgagtgaagctctgctgcacctgcagttcgacgctgatgaggacctgggagctctgctggggaacagcaccgatcccggagtgttcacagatctggcctccgtggacaactctgagtttcagcagctgctgaatcagggcgtgtccatgtctcatagtacagccgaaccaatgctgatggagtaccccgaagccattacccggctggtgaccggcagccagcggccccccgaccccgctccaactcccctgggaaccagcggcctgcctaatgggctgtccggagatgaagacttctcaagcatcgctgatatggactttagtgccctgctgtcacagatttcctctagtgggtctggcagcggcagcgacctttcccatccgcccccaaggggccatctggatgagctgacaaccacacttgagtccatgaccgaggatctgaacctggactcacccctgaccccggaattgaacgagattctggataccttcctgaacgacgagtgcctcttgcatgccatgcatatcagcacaggactgtccatcttcgacacatctctgttt

**---dLbCas12a-Nucleoplamin NLS-VPR**

catttaggtgacactatagataatacgactcactatagggatgagcaagctggagaagtttacaaactgctactccctgtctaagaccctgaggttcaaggccatccctgtgggcaagacccaggagaacatcgacaataagcggctgctggtggaggacgagaagagagccgaggattataagggcgtgaagaagctgctggatcgctactatctgtcttttatcaacgacgtgctgcacagcatcaagctgaagaatctgaacaattacatcagcctgttccggaagaaaaccagaaccgagaaggagaataaggagctggagaacctggagatcaatctgcggaaggagatcgccaaggccttcaagggcaacgagggctacaagtccctgtttaagaaggatatcatcgagacaatcctgccagagttcctggacgataaggacgagatcgccctggtgaacagcttcaatggctttaccacagccttcaccggcttctttgataacagagagaatatgttttccgaggaggccaagagcacatccatcgccttcaggtgtatcaacgagaatctgacccgctacatctctaatatggacatcttcgagaaggtggacgccatctttgataagcacgaggtgcaggagatcaaggagaagatcctgaacagcgactatgatgtggaggatttctttgagggcgagttctttaactttgtgctgacacaggagggcatcgacgtgtataacgccatcatcggcggcttcgtgaccgagagcggcgagaagatcaagggcctgaacgagtacatcaacctgtataatcagaaaaccaagcagaagctgcctaagtttaagccactgtataagcaggtgctgagcgatcgggagtctctgagcttctacggcgagggctatacatccgatgaggaggtgctggaggtgtttagaaacaccctgaacaagaacagcgagatcttcagctccatcaagaagctggagaagctgttcaagaattttgacgagtactctagcgccggcatctttgtgaagaacggccccgccatcagcacaatctccaaggatatcttcggcgagtggaacgtgatccgggacaagtggaatgccgagtatgacgatatccacctgaagaagaaggccgtggtgaccgagaagtacgaggacgatcggagaaagtccttcaagaagatcggctccttttctctggagcagctgcaggagtacgccgacgccgatctgtctgtggtggagaagctgaaggagatcatcatccagaaggtggatgagatctacaaggtgtatggctcctctgagaagctgttcgacgccgattttgtgctggagaagagcctgaagaagaacgacgccgtggtggccatcatgaaggacctgctggattctgtgaagagcttcgagaattacatcaaggccttctttggcgagggcaaggagacaaacagggacgagtccttctatggcgattttgtgctggcctacgacatcctgctgaaggtggaccacatctacgatgccatccgcaattatgtgacccagaagccctactctaaggataagttcaagctgtattttcagaaccctcagttcatgggcggctgggacaaggataaggagacagactatcgggccaccatcctgagatacggctccaagtactatctggccatcatggataagaagtacgccaagtgcctgcagaagatcgacaaggacgatgtgaacggcaattacgagaagatcaactataagctgctgcccggccctaataagatgctgccaaaggtgttcttttctaagaagtggatggcctactataaccccagcgaggacatccagaagatctacaagaatggcacattcaagaagggcgatatgtttaacctgaatgactgtcacaagctgatcgacttctttaaggatagcatctcccggtatccaaagtggtccaatgcctacgatttcaacttttctgagacagagaagtataaggacatcgccggcttttacagagaggtggaggagcagggctataaggtgagcttcgagtctgccagcaagaaggaggtggataagctggtggaggagggcaagctgtatatgttccagatctataacaaggacttttccgataagtctcacggcacacccaatctgcacaccatgtacttcaagctgctgtttgacgagaacaatcacggacagatcaggctgagcggaggagcagagctgttcatgaggcgcgcctccctgaagaaggaggagctggtggtgcacccagccaactcccctatcgccaacaagaatccagataatcccaagaaaaccacaaccctgtcctacgacgtgtataaggataagaggttttctgaggaccagtacgagctgcacatcccaatcgccatcaataagtgccccaagaacatcttcaagatcaatacagaggtgcgcgtgctgctgaagcacgacgataacccctatgtgatcggcatcgataggggcgagcgcaatctgctgtatatcgtggtggtggacggcaagggcaacatcgtggagcagtattccctgaacgagatcatcaacaacttcaacggcatcaggatcaagacagattaccactctctgctggacaagaaggagaaggagaggttcgaggcccgccagaactggacctccatcgagaatatcaaggagctgaaggccggctatatctctcaggtggtgcacaagatctgcgagctggtggagaagtacgatgccgtgatcgcgctagccgacctgaactctggctttaagaatagccgcgtgaaggtggagaagcaggtgtatcagaagttcgagaagatgctgatcgataagctgaactacatggtggacaagaagtctaatccttgtgcaacaggcggcgccctgaagggctatcagatcaccaataagttcgagagctttaagtccatgtctacccagaacggcttcatcttttacatccctgcctggctgacatccaagatcgatccatctaccggctttgtgaacctgctgaaaaccaagtataccagcatcgccgattccaagaagttcatcagctcctttgacaggatcatgtacgtgcccgaggaggatctgttcgagtttgccctggactataagaacttctctcgcacagacgccgattacatcaagaagtggaagctgtactcctacggcaaccggatcagaatcttccggaatcctaagaagaacaacgtgttcgactgggaggaggtgtgcctgaccagcgcctataaggagctgttcaacaagtacggcatcaattatcagcagggcgatatcagagccctgctgtgcgagcagtccgacaaggccttctactctagctttatggccctgatgagcctgatgctgcagatgcggaacagcatcacaggccgcaccgacgtggattttctgatcagccctgtgaagaactccgacggcatcttctacgatagccggaactatgaggcccaggagaatgccatcctgccaaagaacgccgacgccaatggcgcctataacatcgccagaaaggtgctgtgggccatcggccagttcaagaaggccgaggacgagaagctggataaggtgaagatcgccatctctaacaaggagtggctggagtacgcccagaccagcgtgaagcacaaaaggccggcggccacgaaaaaggccggccaggcaaaaaagaaaaaggaaaaggccggcggccacgaaaaaggccggccaggcaaaaaagaaaaagggatgctttagacgattttgacttagatatgcttggttcagacgcgttagacgacttcgacctagacatgttaggctcagatgcattggacgacttcgatttagatatgttgggctccgatgccctagatgactttgatctagatatgctaggaggaagcggaggaggaggtagcggacctaagaaaaagaggaaggtggcggccgctggatccccttcagggcagatcagcaaccaggccctggctctggcccctagctccgctccagtgctggcccagactatggtgccctctagtgctatggtgcctctggcccagccacctgctccagcccctgtgctgaccccaggaccaccccagtcactgagcgctccagtgcccaagtctacacaggccggcgaggggactctgagtgaagctctgctgcacctgcagttcgacgctgatgaggacctgggagctctgctggggaacagcaccgatcccggagtgttcacagatctggcctccgtggacaactctgagtttcagcagctgctgaatcagggcgtgtccatgtctcatagtacagccgaaccaatgctgatggagtaccccgaagccattacccggctggtgaccggcagccagcggccccccgaccccgctccaactcccctgggaaccagcggcctgcctaatgggctgtccggagatgaagacttctcaagcatcgctgatatggactttagtgccctgctgtcacagatttcctctagtgggtctggcagcggcagcgacctttcccatccgcccccaaggggccatctggatgagctgacaaccacacttgagtccatgaccgaggatctgaacctggactcacccctgaccccggaattgaacgagattctggataccttcctgaacgacgagtgcctcttgcatgccatgcatatcagcacaggactgtccatcttcgacacatctctgttt

--Cas9- **Nucleoplamin NLS** -T2A-Cre

atggacaagaagtactccattgggctcgatatcggcacaaacagcgtcggctgggccgtcattacggacgagtacaaggtgccgagcaaaaaattcaaagttctgggcaataccgatcgccacagcataaagaagaacctcattggcgccctcctgttcgactccggggagacggccgaagccacgcggctcaaaagaacagcacggcgcagatatacccgcagaaagaatcggatctgctacctgcaggagatctttagtaatgagatggctaaggtggatgactctttcttccataggctggaggagtcctttttggtggaggaggataaaaagcacgagcgccacccaatctttggcaatatcgtggacgaggtggcgtaccatgaaaagtacccaaccatatatcatctgaggaagaagcttgtagacagtactgataaggctgacttgcggttgatctatctcgcgctggcgcatatgatcaaatttcggggacacttcctcatcgagggggacctgaacccagacaacagcgatgtcgacaaactctttatccaactggttcagacttacaatcagcttttcgaagagaacccgatcaacgcatccggagttgacgccaaagcaatcctgagcgctaggctgtccaaatcccggcggctcgaaaacctcatcgcacagctccctggggagaagaagaacggcctgtttggtaatcttatcgccctgtcactcgggctgacccccaactttaaatctaacttcgacctggccgaagatgccaagcttcaactgagcaaagacacctacgatgatgatctcgacaatctgctggcccagatcggcgaccagtacgcagacctttttttggcggcaaagaacctgtcagacgccattctgctgagtgatattctgcgagtgaacacggagatcaccaaagctccgctgagcgctagtatgatcaagcgctatgatgagcaccaccaagacttgactttgctgaaggcccttgtcagacagcaactgcctgagaagtacaaggaaattttcttcgatcagtctaaaaatggctacgccggatacattgacggcggagcaagccaggaggaattttacaaatttattaagcccatcttggaaaaaatggacggcaccgaggagctgctggtaaagcttaacagagaagatctgttgcgcaaacagcgcactttcgacaatggaagcatcccccaccagattcacctgggcgaactgcacgctatcctcaggcggcaagaggatttctacccctttttgaaagataacagggaaaagattgagaaaatcctcacatttcggataccctactatgtaggccccctcgcccggggaaattccagattcgcgtggatgactcgcaaatcagaagagaccatcactccctggaacttcgaggaagtcgtggataagggggcctctgcccagtccttcatcgaaaggatgactaactttgataaaaatctgcctaacgaaaaggtgcttcctaaacactctctgctgtacgagtacttcacagtttataacgagctcaccaaggtcaaatacgtcacagaagggatgagaaagccagcattcctgtctggagagcagaagaaagctatcgtggacctcctcttcaagacgaaccggaaagttaccgtgaaacagctcaaagaagactatttcaaaaagattgaatgtttcgactctgttgaaatcagcggagtggaggatcgcttcaacgcatccctgggaacgtatcacgatctcctgaaaatcattaaagacaaggacttcctggacaatgaggagaacgaggacattcttgaggacattgtcctcacccttacgttgtttgaagatagggagatgattgaagaacgcttgaaaacttacgctcatctcttcgacgacaaagtcatgaaacagctcaagaggcgccgatatacaggatgggggcggctgtcaagaaaactgatcaatgggatccgagacaagcagagtggaaagacaatcctggattttcttaagtccgatggatttgccaaccggaacttcatgcagttgatccatgatgactctctcacctttaaggaggacatccagaaagcacaagtttctggccagggggacagtcttcacgagcacatcgctaatcttgcaggtagcccagctatcaaaaagggaatactgcagaccgttaaggtcgtggatgaactcgtcaaagtaatgggaaggcataagcccgagaatatcgttatcgagatggcccgagagaaccaaactacccagaagggacagaagaacagtagggaaaggatgaagaggattgaagagggtataaaagaactggggtcccaaatccttaaggaacacccagttgaaaacacccagcttcagaatgagaagctctacctgtactacctgcagaacggcagggacatgtacgtggatcaggaactggacatcaatcggctctccgactacgacgtggatcatatcgtgccccagtcttttctcaaagatgattctattgataataaagtgttgacaagatccgataaaaatagagggaagagtgataacgtcccctcagaagaagttgtcaagaaaatgaaaaattattggcggcagctgctgaacgccaaactgatcacacaacggaagttcgataatctgactaaggctgaacgaggtggcctgtctgagttggataaagccggcttcatcaaaaggcagcttgttgagacacgccagatcaccaagcacgtggcccaaattctcgattcacgcatgaacaccaagtacgatgaaaatgacaaactgattcgagaggtgaaagttattactctgaagtctaagctggtctcagatttcagaaaggactttcagttttataaggtgagagagatcaacaattaccaccatgcgcatgatgcctacctgaatgcagtggtaggcactgcacttatcaaaaaatatcccaagcttgaatctgaatttgtttacggagactataaagtgtacgatgttaggaaaatgatcgcaaagtctgagcaggaaataggcaaggccaccgctaagtacttcttttacagcaatattatgaattttttcaagaccgagattacactggccaatggagagattcggaagcgaccacttatcgaaacaaacggagaaacaggagaaatcgtgtgggacaagggtagggatttcgcgacagtccggaaggtcctgtccatgccgcaggtgaacatcgttaaaaagaccgaagtacagaccggaggcttctccaaggaaagtatcctcccgaaaaggaacagcgacaagctgatcgcacgcaaaaaagattgggaccccaagaaatacggcggattcgattctcctacagtcgcttacagtgtactggttgtggccaaagtggagaaagggaagtctaaaaaactcaaaagcgtcaaggaactgctgggcatcacaatcatggagcgatcaagcttcgaaaaaaaccccatcgactttctcgaggcgaaaggatataaagaggtcaaaaaagacctcatcattaagcttcccaagtactctctctttgagcttgaaaacggccggaaacgaatgctcgctagtgcgggcgagctgcagaaaggtaacgagctggcactgccctctaaatacgttaatttcttgtatctggccagccactatgaaaagctcaaagggtctcccgaagataatgagcagaagcagctgttcgtggaacaacacaaacactaccttgatgagatcatcgagcaaataagcgaattctccaaaagagtgatcctcgccgacgctaacctcgataaggtgctttctgcttacaataagcacagggataagcccatcagggagcaggcagaaaacattatccacttgtttactctgaccaacttgggcgcgcctgcagccttcaagtacttcgacaccaccatagacagaaagcggtacacctctacaaaggaggtcctggacgccacactgattcatcagtcaattacggggctctatgaaacaagaatcgacctctctcagctcggtggagacagcagggctgaccccaagaagaagaggaaggtgaaaaggccggcggccacgaaaaaggccggccaggcaaaaaagaaaaaggaccggtggcagcggagctactaacttcagcctgctgaagcaggctggagacgtggaggagaaccctggacctgccggtTTACTGACCGTACACCAAAATTTGCCTGCATTACCGGTCGATGCAACGAGTGATGAGGTTCGCAAGAACCTGATGGACATGTTCAGGGATCGCCAGGCGTTTTCTGAGCATACCTGGAAAATGCTTCTGTCCGTTTGCCGGTCGTGGGCGGCATGGTGCAAGTTGAATAACCGGAAATGGTTTCCCGCAGAACCTGAAGATGTTCGCGATTATCTTCTATATCTTCAGGCGCGCGGTCTGGCAGTAAAAACTATCCAGCAACATTTGGGCCAGCTAAACATGCTTCATCGTCGGTCCGGGCTGCCACGACCAAGTGACAGCAATGCTGTTTCACTGGTTATGCGGCGGATCCGAAAAGAAAACGTTGATGCCGGTGAACGTGCAAAACAGGCTCTAGCGTTCGAACGCACTGATTTCGACCAGGTTCGTTCACTCATGGAAAATAGCGATCGCTGCCAGGATATACGTAATCTGGCATTTCTGGGGATTGCTTATAACACCCTGTTACGTATAGCCGAAATTGCCAGGATCAGGGTTAAAGATATCTCACGTACTGACGGTGGGAGaATGTTAATCCATATTGGCAGAACGAAAACGCTGGTTAGCACCGCAGGTGTAGAGAAGGCACTTAGCCTGGGGGTAACTAAACTGGTCGAGCGATGGATTTCCGTCTCTGGTGTAGCTGATGATCCGAATAACTACCTGTTTTGCCGGgTCAGAAAAAATGGTGTTGCCGCGCCATCTGCCACCAGCCAGCTATCAACTCGCGCCCTGGAAGGGATTTTTGAAGCAACTCATCGATTGATTTACGGCGCTAAGGATGACTCTGGTCAGAGATACCTGGCCTGGTCTGGACACAGTGCCCGTGTCGGAGCCGCGCGAGATATGGCCCGCGCTGGAGTTTCAATACCGGAGATCATGCAAGCTGGTGGCTGGACCAATGTAAATATTGTCATGAACTATATCCGTAACCTGGATAGTGAAACAGGGGCAATGGTGCGCCTGCTGGAAGATGGCGAT
